# Atherosclerosis nanotheranostics: a perspective from macrophages

**DOI:** 10.7150/thno.121239

**Published:** 2026-09-02

**Authors:** Caiyan Zhao, Jiakun Zhang, Rujie Wang, Shen’ao Wang, Hongzhang Deng

**Affiliations:** 1 School of Life Science and Technology, Xidian University & Engineering Research Center of Molecular and Neuro Imaging, Ministry of Education, Xi'an, Shaanxi 710126, China.; 2 Institute of Biotechnology and Food Science, Hebei Academy of Agriculture and Forestry Sciences, Shijiazhuang 050051, China.; 3 International Joint Research Center for Advanced Medical Imaging and Intelligent Diagnosis and Treatment & Xi'an Key Laboratory of Intelligent Sensing and Regulation of trans-Scale Life Information, School of Life Science and Technology, Xidian University, Xi'an, Shaanxi 710126, China.

**Keywords:** atherosclerosis, macrophages, nanomaterials, diagnosis, treatment

## Abstract

The mortality rate of cardiovascular diseases (CVDs) has ranked number one in the world over the years. Atherosclerosis (AS), a chronic inflammatory disease, is a key pathological factor in the occurrence and development of CVDs, characterized by lipid accumulation and plaque formation in arteries. Macrophages is the critical contributor to the progression of AS, and macrophage-related pathological processes have received considerable attention as an extremely important target for the diagnosis and treatment of AS. Biomaterial-based nanotechnology offers the possibility of selective and efficient delivery of therapeutic agents to atherosclerotic macrophages, shows the potential to improve the understanding of the pathophysiology of AS, contributes to improve the therapeutic outcomes and imaging accuracy, and further minishes the risk of CVDs. This review provides a well-rounded snapshot of the features, functions, and critical roles of macrophages in the development and progression of AS, as well as the recent advances in the application of macrophage-related nanomaterials in the diagnosis and treatment of AS, with specific emphasis on the design and integration of nanomaterials for specific macrophages-manipulating. Finally, the challenges and prospects of macrophage-based nanotechnologies for the prevention and treatment of AS are also discussed in depth, aiming to inspire innovative thinking and facilitate the ever-fast development of nanomedicine for AS.

## 1. Introduction

With the rapid development of society and the economy and the gradual improvement of people's daily living standards, the morbidity and mortality rates caused by cardiovascular diseases (CVDs) have been far higher than those caused by cancer and other major diseases. Atherosclerosis (AS) is the main pathophysiological basis of various acute CVD events, such as coronary heart disease, myocardial infarction and ischemic stroke, and has become the first "killer" threatening the safety of human life 1. The pathogenesis of AS is extremely complex, and the main characterization is the formation of vulnerable plaques beneath endothelium. Rupture of vulnerable plaques and subsequent thrombosis are the precipitating factors for related fatal diseases, which are greatly associated with the expansion of necrotic lipid-rich cores, thinning of fibrotic caps, infiltration of inflammatory cells, and so on 2. What’s more, the arteries are located in the deep parts of an organism, so there will be hidden dangers in arteries everywhere, such as the aorta, coronary artery, and cerebral artery. Hence, AS remains a recognized research hotspot and challenge in the cardiovascular field.

The occurrence and progress of AS is mainly mediated by complicated inflammation and immune responses. As a chief inflammatory cell, macrophage plays a pivotal role in the development of AS 3. Specifically, the oxidized phospholipids and cholesterol in lipoproteins that retained in the lesion areas induce endothelial cells activation, which further triggers the recruitment of monocytes. In the intima of the artery, monocytes are transformed into macrophages. Under the stimulation of local cytokines, macrophages differentiate into macrophage subtypes exercising different functions, which collectively participate in the pathogenesis of AS and play different roles in the evolution of AS 4. Macrophages phagocytize and clear apoptotic cells through efferocytosis, and eliminate damaged organelles and misfolded proteins by inducing autophagy, thus enhancing macrophage-derived cholesterol efflux and inhibiting the occurrence and development of AS 4. Moreover, there is evidence that macrophages are involved in the remodeling of the atherosclerotic microenvironment. The plaque microenvironment is a highly complex physicochemical environment, showing weakly acidic (pH 6.0-6.8), and enriched with inflammatory factors, chemokines, reactive oxygen species (ROS), enzymes, and lipids 6, 7. Consequently, appropriate therapeutic strategies by taking advantage of the multiple roles of macrophages are needed to effectively reduce the plaque inflammatory response and delay the progression of AS from the perspective of pathological mechanism, which is therefore crucial to overcome the unpleasant dilemma of AS.

The application of nanomaterials in the diagnosis and treatment of AS and related diseases have been extensively studied and shown revolutionary effects 8. Due to their inherently small size, the nanoparticles can effectively evade the clearance mechanisms in the body, prolong systemic circulation, and improve pharmacokinetic profile 9. Some nanomaterials also exhibit unique physicochemical properties, such as optical, thermal, magnetic, and catalytic properties, contributing to disease-specific diagnosis and treatment 10, 11. Through rationally design, the nanomaterials can also take full advantage of the physiological and pathological characteristics of the atherosclerotic lesion to achieve ideal permeability and retention 12. Moreover, nanomaterials can be functionalized by modifying the surface with specific ligands to target inflammatory macrophages enriched in atherosclerotic lesions, which provides more possibilities to realize precise, efficient, and personalized diagnosis and treatment for AS 13, 14.

Although the previous literatures have summarized several macrophage-based treatment strategies and imaging technologies for AS, to the best of our knowledge, there is no systematic review of the application of macrophage-targeting nanotechnologies in the treatment and diagnosis of atherosclerotic plaques from the perspective of nanomaterials. In this review, we expand on available work and highlight recent advances in state-of-the-art nanomaterials for the treatment and diagnosis of AS (Figure 1). To pursuit this goal, the origin, phenotype, function of macrophages, and their influences on the shaping of plaque microenvironment in the genesis and development of atherosclerotic disease were elaborated first, and the potential targets for treatment and diagnosis were introduced. Subsequently, the mechanism of macrophage-targeted nanomaterials for drug delivery were discussed. The various nanomaterials and their application are mainly discussed with the most recent research outcomes in macrophage-based AS management. Finally, the future challenges and prospects facing anti-AS therapy are also prospected.

## 2. The pathophysiologic characteristics and functions of macrophages in AS

The onset and progression of AS are associated with localized inflammation and lipid accumulation in the vascular wall. Although various cells participate in the formation and progression of AS, macrophages remain central players. Macrophages play a critical role in the advancement of AS and are the most commonly studied target because of the pathogenic roles, phagocytic property and abundance in the AS lesion. Research targeting macrophage may open new therapeutic avenues for the prevention and treatment of cardiovascular diseases such as atherosclerosis.

### 2.1. Differentiation and regulation of macrophages

In atherosclerotic lesions, macrophages are regulated by various microenvironmental signals such as oxidized lipids and cytokines. Macrophages in AS are mainly derived from monocytes that migrate to the intima of blood vessels *via* the recruitment of chemokines and then polarize and reprogram in response to different stimuli (Figure 2A).

Macrophage polarization refers to the process by which macrophages differentiate into diverse phenotypes due to microenvironmental stimulation including chemokines, cytokines, and growth factors 7. Monocyte colony-stimulating factor (M-CSF) and granulocyte-monocyte colony-stimulating factor (GM-CSF) are the key regulators of macrophage differentiation induction. M-CSF induces the production of anti-inflammatory M2 macrophages in early AS lesions, while GM-CSF promotes the polarization of proinflammatory M1 macrophage phenotypes during AS development 15. A study has shown that Th1 and Th2 cells in AS lesions can also release macrophage polarization-related factors to affect the balance of M1 and M2 phenotypes 16. M1 macrophages mainly secrete a mass of proinflammatory factors, including TNF-*α*, IL-1*β*, IL-6, and NO synthase, to promote and sustain inflammation 17. M1 macrophages also produce reactive oxygen species and nitric oxide in an effort to clear viral, bacterial, and fungal infections. The distinct chemokine (C-X-C motif) receptor ligands (CXCL9, CXCL10, and CXCL5) expressed by M1 macrophages further promote the recruitment of Th1 and natural killer cells, which play crucial roles in eliminating intracellular pathogens. Although these effects are beneficial during acute infections, the chronic induction of M1 macrophages can lead to tissue damage and impede wound healing, particularly under sterile inflammatory conditions. M2 macrophages can be further subdivided into M2a, M2b, M2c, and M2d subtypes 18. The M2a phenotype is triggered by IL-4 and IL-13, highly expresses the mannose receptor (MR or CD206), and secretes pro-fibrotic factors such as fibronectin, insulin-like growth factor (IGF), and transforming growth factor beta (TGFβ), which promote tissue repair. The M2b phenotype is induced upon simultaneous exposure to immune complexes combined with Toll-like receptor (TLR) agonists or IL-1 receptor agonists. While M2 macrophages are generally considered anti-inflammatory, characterized by high IL-10 and low IL-12 expression, M2b macrophages appear to be an exception. They concurrently produce high levels of pro-inflammatory cytokines (IL-1β, IL-6, and TNF) along with IL-10, while expressing low levels of IL-12. M2c macrophages are induced by IL-10 and glucocorticoids. They highly express Mer receptor tyrosine kinase (MerTK) and possess a strong capacity for efferocytosis. The M2d phenotype is induced by TLR agonists that stimulate the adenosine A2A receptor. These M2d macrophages are characterized by high expression of IL-10 and vascular endothelial growth factor (VEGF), endowing them with pro-angiogenic properties 19. The proportion of M1/M2 macrophages could reflect the stability of plaques to some extent. Beyond M1 and M2 phenotypes, additional phenotypes of macrophages have also been found in atherosclerotic lesions, including hemorrhage induced macrophages M(Hb), phospholipid-triggered Mox macrophages, and platelet-derived chemokine CXCL4-activated M4 macrophages. After phagocytosing the hemoglobin-haptoglobin complex, macrophages form M(Hb) macrophages, which highly express the M2 markers MR and CD163 on their surface. M(Hb) can reduce lipid accumulation and ROS production 20. Boyle *et al*. identified a novel macrophage subpopulation located in plaque hemorrhage areas-termed HA-mac-characterized by high expression of CD163 but low expression of human leukocyte antigen-DR (HLA-DR) 21. These macrophages primarily exert anti-atherosclerotic effects by participating in hemoglobin clearance and reducing oxidative stress. Closely related to HA-mac are Mhem macrophages, which possess a robust capacity for erythrophagocytosis and exhibit strong phosphorylation-dependent activation of the transcription factor nuclear factor erythroid 2-related factor 1 (Nrf1). This transcription factor induces the expression of heme oxygenase-1 (HMOX-1) and activates the liver X receptor α (LXRα)/ABCA1/apolipoprotein E (ApoE) cascade, thereby exerting a protective effect by inhibiting foam cell formation. Mox macrophages show the enhanced expression of antioxidant-related genes and reduced phagocytosis 22. Based on the expression of matrix metalloproteinase 7 (MMP7) and the calcium-binding protein S100A8, a population of M4 macrophages has been identified in human atherosclerotic plaques. These cells co-express MMP12, the mannose receptor, and pro-inflammatory factors such as IL-6 and TNF-*α*. As a hallmark feature of CXCL4 polarization, M4 macrophages do not express CD163, the scavenger receptor for the hemoglobin-haptoglobin complex. The phenotype of M4 macrophages is similar to that of M1; but they don't possess the phagocytic capability 23.

Macrophages play a central role throughout the stages of AS (Figure 2B). In the early stage of AS, recruited monocytes differentiate into macrophages and phagocytize oxidized low-density lipoprotein (OX-LDL) to form foam cells. Foam cells are the main sources of plaques in AS 24. At early stage, the foam cell formation and the efferocytotic capacity of M2 macrophages are stronger than those of M1 macrophages, thus promoting the regression of inflammation and inhibiting the progression of AS 25. In the developmental stage of AS, apoptotic cells cannot be effectively cleared due to the defective efferocytosis of macrophages, resulting in mass aggregation and necrosis, which eventually leads to the continuous expansion of the necrotic core and causes an inflammatory cascade reaction. In the later stage of AS development, macrophages secrete MMPs to degrade fibrous tissue, resulting in fiber cap thinning and advanced plaque instability 26. Fragile plaques are prone to rupture, which can lead to blood clots and serious CVDs 27.

The quantities of both M1 and M2 macrophages increase with the progression of atherosclerotic disease, and the abundance of both phenotypes is higher in rupture-prone unstable plaques than in stable plaques. Different macrophage subtypes exhibit distinct spatial distributions within plaques: M1 macrophages are predominantly located in the vulnerable shoulder regions, showing no significant predominance in the fibrous cap. In the fibrous cap area, the pro-fibrotic and tissue-repair functions of M2 macrophages may counteract the detrimental effects of M1 macrophages. However, in the plaque shoulders, the limited number of M2 macrophages is insufficient to balance the M1-mediated pro-inflammatory effects. Surprisingly, a significant infiltration of M2 macrophages is observed in the adventitial layer 28. Recent studies comparing specimens from patients with acute ischemic events and asymptomatic individuals revealed that M1 macrophages are present only in the plaques of symptomatic patients and are more abundant in unstable plaques. M2 macrophages are found in both patient groups but exhibit a higher proportion in stable plaques. In human atherosclerotic lesions, Mhem macrophages are densely distributed in areas of previous hemorrhage, neovascularization, and iron deposition. Intraplaque hemorrhage accelerates lesion progression through the dual stimulation of cholesterol-rich erythrocyte membranes and oxidized heme/iron. Surrounding macrophages can polarize into the Mhem phenotype-which, despite its high iron content, exhibits minimal oxidative damage. The high expression of IL-10, heme oxygenase-1 (HO-1), and enhanced capacity for lipid efflux establish this as an anti-atherosclerotic phenotype. In advanced atherosclerotic lesions of low-density lipoprotein receptor knockout mice, Mox macrophages account for up to 30% of the total macrophage population. These cells alleviate oxidative stress by highly expressing antioxidant enzymes such as heme oxygenase-1 (HMOX1) and thioredoxin reductase 1 (Txnrd1). Their ability to modulate the plaque's redox status, coupled with their characteristic suppression of phagocytic activity, collectively influences the trajectory of the disease 29.

Understanding of the differentiation and regulation of macrophages in the whole stages of AS has important potential value for the treatment of AS. Based on many related studies, several strategies, including depolarizing and modulating macrophages, enhancing efferocytosis, inducing proinflammatory macrophage apoptosis, and anti-inflammatory approaches, have been proposed as promising therapies 30, 31.

### 2.2. Engulfing low density lipoproteins

Macrophages are phagocytic cells, and they are able to internalize dying or dead cells and foreign bodies. Additionally, after migrating from the vascular media to intima, the macrophages take up lipoproteins. These lipoproteins are subsequently retained and modified through various processes, including efferocytosis, micropinocytosis, phagocytosis, and scavenger receptor-mediated uptake. Such modifications represent beneficial changes that help clear inflammatory lipids. However, excessive lipid uptake by macrophages promotes their transformation into foam macrophages, which marks the initiation of AS 32. At this stage, free cholesterol-induced macrophage apoptosis further exacerbates the expansion of chronic inflammation. During this process, the involved macrophage scavenger receptors (SRs) include SR-A, CD36 receptor, lectin-like oxidized LDL receptor-1 (LOX-1), SR-B1, CD68 receptor, macrophage receptor with collagenous structure (MARCO) receptor and so on (Figure 2B) 33. Among them, the subtype SR-A and CD36 are two primary receptors. They are also involved in the progress of atheroinflammation. SR-A receptor is highly expressed on plaque macrophages but not found in normal vessel walls 34. Thus far, various approaches to target the SR-A of macrophages have been explored in AS diagnosis and therapy. CD36 is an 88 kDa transmembrane receptor, which is more important than other macrophage scavenger receptors in transporting ox-LDL as well as facilitating the formation of foam cell 35. It also correlates well with the severity of atherosclerotic lesions.

Studies have demonstrated that SR-A can mediate the proliferation of macrophages within plaque, while CD36 can inhibit macrophage migration and promote their spreading and adhesion, as well as coordinate inflammasome activation. Moreover, SR-A and CD36 collectively promote cell apoptosis, expansion of the necrotic core in plaques, and the expression of inflammatory gene. Notably, these latter effects persist even when plaque area and foam cell formation remain unaffected 4. Therefore, in addition to facilitating lipid uptake, SR-A and CD36 appear to possess important signaling functions that influence the progression of atherosclerosis.

Motivated by the negative charge on ox-LDL, polyanionic macromolecules such as malondialdehyde modified LDL, maleylated bovine serum albumin, and polyinosinic acid have been proposed for the target of SR-A receptor. What is notable is the dextran sulfate (SDIO) that can be attached to nanoparticles for the specifical target of SR-A 36, 37. In addition, the binding of macrophage epsin, a family of endocytic adaptors, to CD36 can enhance the endocytosis and recycling of CD36, thus promoting lipid uptake 38.

### 2.3. Promoting cholesterol efflux

To avoid lipid deposition in the cytoplasm, overproduced cholesterol can be transported out of macrophages and undergoes degradation in liver. This process involves three important macrophage membrane proteins: ATP-transporter cassette A1 (ABCA1), ATP-transporter cassette G1 (ABCG1) and SR-B1 39. ABCA1/G1 facilitates the efflux of free cholesterol from macrophages/foam cells to pre-β-HDL that is composed of apolipoprotein AI (apoA-1) and phospholipids 40. Subsequently, free cholesterol on pre-*β*-HDL is esterified by lecithin cholesterol acyltransferase (LCAT) to cholesteryl ester that is then transported into the hydrophobic core of HDL. When more and more cholesterol is picked up from peripheral cells, the accumulation of cholesteryl ester in HDL expands the size of HDL and converts it into mature HDL 41. Then hepatocytes selectively take up cholesteryl ester in mature HDL through apoA-1-mediated binding of cholesteryl ester to hepatocyte SR-B1 receptor 42. The absorbed cholesteryl ester in hepatocytes can be utilized to synthesize bile acid and then excretes into bile. If the intestine cannot reabsorb cholesterol and bile acid, they are cleaned up into the feces. Deficiencies in ABCA1 and ABCG lead to inflammation in macrophages and promote the development of atherosclerosis. Studies have shown that the cholesterol efflux capacity of high-density lipoprotein is inversely correlated with the incidence of cardiovascular events in humans. One of the primary functions of ABCA1 and ABCG1 is to prevent the accumulation of free cholesterol in bone marrow hematopoietic stem cells, thereby inhibiting their proliferation and subsequent leukocytosis in mice following a high-fat diet. Moreover, these transporters almost certainly play a crucial local role in macrophages at lesion sites 4. Thus, dysregulation of cholesterol metabolism may contribute to atherosclerosis by affecting various inflammatory signals and detrimental processes in macrophages. Conversely, inflammation can suppress cholesterol efflux from macrophages and reverse cholesterol transport in the body. The expression of macrophage membrane proteins is induced by the cholesterol-sensing nuclear receptors LXR. LXR*α* and LXR*β* are both expressed in macrophages. The activation of LXR enhances macrophage cholesterol efflux, thereby reversing AS, ameliorating inflammatory response and promoting efferocytosis. Studies focusing on LXR have confirmed the positive role of cholesterol efflux in the regression of atherosclerosis. As a transcription factor, LXR induces the expression of genes involved in cholesterol transport and efflux, which is critical for delaying the progression of atherosclerosis and promoting its regression. Additionally, the nonspecific efflux molecule cyclodextrin can facilitate atherosclerosis regression through LXR-mediated macrophage reprogramming, with mechanisms including enhanced cholesterol efflux and anti-inflammatory effects 43. GW3965 is one of the classical LXR agonists 44. Ghosh *et al*. designed a mannose-decorated dendrimeric nanoparticle (mDNP) to simultaneously deliver LXR ligand and SR-A siRNA to plaque macrophage to decrease macrophage lipid conten 45. Similarly, Fisher *et al*. developed a vesicle formed by PLGA-*b*-PEG block copolymers to deliver GW3965 46. Schwendeman attempted to use synthetic HDL (sHDL) nanoparticles to deliver the LXR agonist, T0901317 (T1317), to promote cholesterol efflux from macrophages and reduced liver hypertriglyceridemia side effects 47.

## 3. The role of macrophages in shaping the microenvironment of AS

In the progression of AS, macrophages, as a central element, play a crucial role in shaping the atherosclerotic plaque microenvironment, including secreting chemokines, releasing inflammatory cytokines to induce an inflammatory microenvironment; phagocytosing ox-LDL, converting anaerobic glycolytic metabolism to decreased pH value in the plaque microenvironment; generating ROS and NO through the NADPH oxidase system; and producing excess proteases to destabilize atherosclerotic plaques, erode the fibrous cap, and increase the risk of plaque rupture (Figure 2B) 48.

### 3.1. Inflammatory microenvironment

Chronic inflammation is one of the main features of AS 15. During the progression of AS, macrophages at the lesion site are able to activated by oxLDL through toll-like receptors and NF-kB nuclear translocation 49. oxLDL is recognized by CD14-TLR4-MD2, and this interaction induces cytoskeletal rearrangement and promotes the production of TNF-a, IL-6, and IL-10 50. Besides oxLDL, cholesterol crystals can activate the NACHT, LRR, and PYD domain protein 3 (NLRP3) inflammasome in foam cells, causing the release of IL-1β 51, 52. M1 phenotype of macrophages also secrete numerous chemokines and other proinflammatory cytokines. These cytokines play a vital role as immune recognition receptors in inducing the acquired immune response pathway, participate in the activation of T lymphocytes and amplify the local inflammatory response by recruiting T lymphocytes to the intima as well as secreting TNF-*α* and INF-*γ* 53. C-C motif ligand 2 and its receptor CCR2 represent the earliest identified chemokine-receptor unit that demonstrated to participate in the initiation of atherosclerosis in mice by promoting monocyte recruitment to lesion sites. The CCR2 has also been confirmed to exhibit pro-atherosclerotic effects 4. IL-1α and IL-1β act as atherogenic agents that increase the expression of adhesion molecules and activate macrophages. IL-1β is a crucial Th17 cell differentiation factor that can enhance inflammation in the vascular wall. The expression of IL-18 is elevated in atherosclerotic plaques as well as in samples from patients with diabetes mellitus and myocardial infarction. The function of TNF-α in atherosclerosis is associated with endothelial cell activation, leading to increased expression of various adhesion proteins, promoting the production of other pro-inflammatory cytokines, and recruiting immune cells to the affected arterial intima. M1 macrophages produce CD40. The levels of CD40 and CD40L in plaques are directly related to vascular remodeling 54. Persistent inflammation within plaques is a key factor in the rupture of vulnerable atherosclerotic plaques. Currently, a number of nanocarriers have been developed to target the sites of inflammation by the delivery of anti-inflammatory drugs to reduce anti-inflammatory responses in high-risk plaques 55.

### 3.2. Acidic microenvironment

After migrating to the intima, macrophages engulf ox-LDL to form foam cells, marking the onset of AS. This process is energy-dependent and extremely requires the consumption of adenosine triphosphate (ATP) 56. Lipid overload forces macrophages to switch from aerobic metabolism to anaerobic glycolysis to generate energy, resulting in the uncontrolled accumulation of lactate and a drop in pH in the microenvironment of atherosclerotic plaques 57. Naghavi *et al*. employed two pH-sensitive fluorescent probes to measure pH in atherosclerotic plaques 58. They found that the pH ranged in the interstitial microenvironment in AS lesions of human and rabbit were approximately 6.5-8.5 and 5.5-7.5, respectively. In addition, lysosomes in cells are acidic, including those of macrophages. They have a pH range of approximately 4.7-4.8 59, 60. Taking advantage of these properties, Researchers developed a serial of pH-sensitive nano delivery system for the AS therapy 61.

### 3.3. Oxidative microenvironment

ROS are a group of free radicals and reactive nonradical molecules including superoxide (O_2_^.-^) radicals, hydrogen peroxide (H_2_O_2_), hydroxyl (HO^.^) radicals, and peroxynitrite (ONOO^-^) etc 62. Normal physiological levels of ROS are critical for vascular homeostasis. Overproduction of ROS could lead to vascular damage. Some risk factors for AS (e.g. hypercholesterolemia) can mediate the overproduction of ROS through the involvement of several prooxidant systems, including mitochondrial oxidative stress and the reduced form of nicotinamide adenine dinucleotide phosphate oxidase. Macrophages play a critical role in the regulation of ROS metabolism. Activated M1 macrophages can generate ROS and nitric oxide (NO) by activating the NADPH oxidase system, thereby modulating their phagocytic function, disrupting normal cellular metabolism, inducing apoptosis, and ultimately leading to chronic tissue damage and plaque formation. Additionally, the pentose phosphate pathway exhibits a dual role in regulating cellular redox status: by generating nicotinamide adenine dinucleotide phosphate (NADPH), this pathway supports various antioxidant systems, which is crucial for protecting cells from oxidative damage. Simultaneously, NADPH oxidase also utilizes the NADPH produced by this pathway to generate reactive oxygen species. During the polarization of macrophages toward the M1 phenotype, enhanced glycolysis is accompanied by increased activity of the pentose phosphate pathway, which plays a decisive role in sustaining the inflammatory response of macrophages 63. Based on the adverse effects caused by the overproduction of ROS, researches focus on the ROS-scavenging antioxidant therapy for the exploration of a reasonable and potential strategy to alleviate AS 64, 65.

### 3.4. Over-expressed proteases

In an inflammatory state, activated macrophages release multiple proteases, such as cysteine proteases and MMPs, into the biological microenvironment of plaques to degrade extracellular matrix, erode fibrous cap, and increase the risk of plaque rupture 66. Macrophages promote arterial expansive remodeling by degrading extracellular matrix proteins through increased production of MMPs, particularly MMP-2 and MMP-9. Plaques with thin fibrous caps may detach from the endothelium, potentially triggering thromboembolic events. Therefore, macrophage-derived MMPs play a critical role in remodeling the extracellular matrix and cell surfaces, significantly influencing the ultimate outcome of atherosclerotic cardiovascular diseases. M1 macrophages upregulate the expression of MMP-1, MMP-3, MMP-10, MMP-12, MMP-14, and MMP-25 via mechanisms dependent on mitogen-activated protein kinase (MAPK) and phosphatidylinositol 3-kinase (PI3K). Various chemokines, such as TNF-α, GM-CSF, and IL-1β, can regulate MMP activity through both prostaglandin-dependent and -independent pathways. In contrast, M2 macrophages downregulate the expression of MMP-2, MMP-8, and MMP-19 while upregulating MMP-11, MMP-12, MMP-25, and tissue inhibitor of metalloproteinase-3. The differential regulation of MMP expression by M1 and M2 macrophages offers a potential avenue for modulating the stability of atherosclerotic plaques 28. Cathepsins, another class of proteolytic enzymes secreted by macrophages, can enhance the inflammatory activity of AS. As a distinct feature of the atherosclerotic plaque microenvironment, increased proteases have become a promising stimulus for the development of atherosclerotic drug delivery and imaging probes. Different macrophage related proteases-sensitive peptides can be modified on the nanocarriers according to the microenvironment of the AS, to target the lesions 67.

## 4. Nanomaterials targeting atherosclerotic macrophages for AS therapy

### 4.1. Nanomaterials for targeted drug delivery to macrophage

Nanotechnology has revolutionized the field of biomedicine. Through the ingenious design and use of nanomaterials, nanomedicines offer efficient, precise, safe, and personalized approaches for the treatment and diagnosis of diseases, and show great promise to reverse the barriers in currently available strategies. Several nanomedicine platforms have been prepared to target atherosclerotic macrophages 68. Understanding the process by which nanomedicine reached macrophages will contribute to design nanomedicines to better target plaque macrophages, thus improving the therapeutic effect of AS.

With their inherently reduced size (1-1000 nm), most nanomaterials can be efficiently distributed in the body through blood circulation, successfully minimize internal clearance mechanisms and prolong blood circulation, which improves the bioavailability of therapeutic agents 69. During blood circulation, adsorption of biomolecules on the surfaces of nanomaterials can affect its biodistribution, pharmacokinetics and cellular uptake. For instance, adsorption of opsonin proteins (e.g. IgG) leads to the removal of nanomedicines by mononuclear phagocyte system (MPS) in the liver and spleen 70. PEGylation of the surface of nanomaterials is the most extensive approach to evade MPS recognition and prolong their circulation time. However, this method has been associated with the potentially toxic effects of anaphylaxis, which may trigger anaphylactic shock 71. Surface coating of nanomedicines with cell membranes (*e.g.*, erythrocyte membrane, platelet membrane, macrophage membrane, stem cell membrane, and neutrophilic cell membrane) is an alternative strategy to induce immune escape. And it also provides additional biological properties for nanomaterials, such as selective adhesion, inflammatory site targeting, and endothelial penetration.

#### 4.1.1. Passive target

Nanomaterials have a certain ability to passively target atherosclerotic macrophages. When the plaque grows, the connectivity of the endothelial cells covered by the atherosclerotic plaque is significantly reduced, the endothelial barrier is destroyed, and the permeability of the endothelial layer of the arterial wall is increased 72. A high amount of nanomaterials are accessible to intimal layer from areas with increased membrane permeability. In addition, vascular lesions more prefer to evolve at sites characterized by disturbed blood flow and lower shear stress at the walls, where nanomaterials also tend to be deposited 73. The sizes of nanomaterials play an important role in their accumulation in plaques. It has been found that most nanoparticles larger than 7 nm are readily trapped by capillaries. The nanomedicines with diameters ranging from 7 to 30 nm have a better retention effect than those larger than 70 nm 74. What should be noted is that the macrophages in the lesion sites show strong phagocytosis. Apoptotic bodies, nanomaterials or other foreign bodies can be ingested by macrophages. Nanoparticles have the chance to be taken up by circulating monocytes through phagocytosis, and eventually accumulate in the atherosclerotic plaques 75. Zhang *et al*. designed a unique tetrapod needle-like PdH nanoenzyme, which achieves efficient treatment of atherosclerosis by synergistically scavenging reactive oxygen species (ROS), exerting the anti-inflammatory effects of hydrogen, and activating autophagy. After being internalized by macrophages, these tetrapod needle-like PdH nanoenzymes leverage the macrophages' high-efficiency chemotactic sensing capability to precisely target inflammatory sites. They effectively reduce ROS levels, significantly suppress inflammation-related pathological processes, and demonstrate remarkable antioxidant and anti-inflammatory properties, thereby alleviating the progression of atherosclerosis 76.

#### 4.1.2. Active target

Active targeted modification of the surface of nanomedicines also improved their accumulation at the lesion sites. More importantly, active targeted modification enhanced the internalization of nanomedicines by lesioned macrophages, which is the key to improve therapeutic efficacy as numerous nanomedicines need to enter into cells to function 77. Apart from above mentioned scavenger receptors, other macrophage-specific surface receptors, such as folate receptor beta (FR-β) and mannose receptor, can also be attached to nanomaterials for the purpose of targeting intimal macrophage (Figure 3A). It has been acknowledged that FR-β is overexpressed in activated macrophages in vulnerable plaques but it is not found on the surfaces of non-activated macrophages or other immune cells 78. This meaningful receptor can be exploited for the treatment of vulnerable plaques. For instance, the poly(ethylene glycol)-coated, acetic-anhydride-capped, and folate ligand-functionalized PAMAM dendrimers (Fol-Dend) were created by Low and coworkers for the investigation of their application in anti-AS and ulcerative colitis treatment. Their studies investigated that the folate decorated dendrimers were specifically uptaken by folate receptor-positive macrophage cells and selectively deposited in the inflammation site in mouse models of AS. A clear Fol-Dend-Cy5.5 signal was observed in the inflammatory regions of high-fat diet-fed mice, while only minimal accumulation of non-folate-functionalized NT-Dend-Cy5.5 was detected in the aortas of atherosclerotic mice 79. Mannose receptor (MR, CD206), an extremely effective endocytic C-type lectin receptor, is another interesting target for the treatment and imaging of vulnerable plaques because it is highly expressed on macrophages at the sites of fibrous cap of plaques but is rarely expressed on apoptotic macrophages located in the lipid core 80. In this concern, a Gallium-68 (^68^Ga)-labelled anti-MR nanobody (^68^Ga-anti-MMR Nb) has been reported to visualize the atherosclerotic plaques through non-invasive PET/CT imaging. Positively, in vitro studies indicated that ^68^Ga-anti-MMR Nb specifically bond to M2a macrophages. *In vivo* studies demonstrated the focal signals of ^68^Ga-anti-MMR Nb in aortic tissue of ApoE-KO mice. And the deposition of ^68^Ga-NOTA-anti-MMR Nb was observed in atherosclerotic plaques on autoradiographs. The mean percentage of tracer uptake in the intact aortas dissected from ApoE knockout non-blocked mice was more than 5 times greater than that in the control group 81. Zhang *et al*. reported a nanoparticle-mediated mRNA therapy that targets macrophages within atherosclerotic plaques. The nanoparticles were constructed through microfluidic self-assembly using a cationic lipid molecule (G0-C14) and poly(lactide-co-glycolide)-b-poly(ethylene glycol) (PLGA-PEG) copolymer. The surfaces were then functionalized with mannose to confer macrophage-targeting capability to the G0-C14/PLGA-PEG nanoparticles (M-HNPs). In Ldlr-/- mice, the Cy5-labeled, IL-10 mRNA@M-HNPs exhibited approximately 3-fold higher fluorescence signal intensity in aortic tissues compared to the non-functionalized IL-10 mRNA@HNPs-treated group. Concurrently, plaque inflammation was significantly alleviated, as evidenced by reduced oxidative stress and macrophage apoptosis, along with a decrease in necrotic areas 82.

Activated macrophages in the in vulnerable plaques of AS have also been reported to greatly express transferrin receptor 1 (TfR1). High expression of TfR1 is closely correlated with the infiltration of macrophages, the development of atherosclerotic lesions, and the risk of plaque rupture 83. Human heavy-chain ferritin (HFn) can specifically bind to infiltrated macrophages within atherosclerotic plaques via TfR1. Significantly improved level of HFn protein has been detected in the infiltrated macrophages in human atherosclerotic plaques. Using this characteristic, Yan and coworkers radiolabeled natural H-ferritin nanocages with technetium-99m (^99^mTc-HFn) to monitor of the progression of AS by combined single-photon-emission computed tomography (SPECT) and computed tomography (CT) imaging. The results indicate that the binding constant (Kd) of HFn to plaque macrophages is 54.2 nM, with a maximum binding capacity (Bmax) of 34.6 × 10⁻¹⁹ mol/cell, demonstrating high binding affinity and capacity of HFn for plaque-infiltrated macrophages. Their probes could quantitatively assess the dynamic changes of inflammation during the progression of AS plaques and guide the anti-inflammation treatment (Figure 3B-C) 84. HA receptors and Fc receptors are also highly expressed on the surfaces of macrophages. Therefore, attaching their ligands to the nanomaterials is also a promising approach for effective targeting of the fibrous cap of AS 14.

### 4.2. Macrophages targeted nanomaterials for AS therapy

#### 4.2.1. PLGA based nanomaterials

PLGA is the FDA-approved copolymer composed of lactic and glycolic acids. Benefited from its unique physicochemical characteristics, biodegradablity, biocompatibility, and easy synthesis, PLGA has become a major class of controlled release systems. The advantage of PLGA formed colloidal nanoparticles in AS therapy is effective delivery of its contents to atherosclerotic lesions without their adverse effects 46, 85. As an example, Egashira *et al*. prepared the PLGA nanoparticle to deliver pitavastatin, which effectively decreased the recruitment of inflammatory Ly-6Chigh monocytes to atherosclerotic plaques, leading to the reduction of macrophage content in the atherosclerotic plaque and the inhibition of plaque rupture 86. They further used the PLGA nanoparticles-mediated delivery of pioglitazone, a potent peroxisome proliferator–activated receptor-γ (PPARγ) agonist, to modulate the polarity of macrophages, which resulted in the enhanced activation of alternative (M2) phenotypes with the expression of anti-inflammatory factors 87. Particularly, the PLGA nanoparticle can be readily functionalized by chemical modification to target deliver loaded therapeutics to specific disease sites and/or achieve intelligent responsiveness 82. Based on the tissue factor (TF) protein on the surfaces of atherosclerotic macrophages, Liao *et al*. investigated the feasibility of EGFP-EGF1-coupled PLGA nanoparticles (ENPs) as a carrier for CCR_2_-shRNA targeted delivery to atherosclerotic macrophage. ENPs can effectively enter into atherosclerotic macrophages by adhering to the TF protein on the surface of macrophage. Therefore, they can act as effective carriers to induce the CCR2 gene silence in the atherosclerotic macrophages and contribute to reduce macrophage recruitment for the therapy against AS (Figure 4A) 88. Several PLGA-based carriers for gene delivery to atherosclerotic macrophages have also caught special attention. They can effectively protect the contents from degradation, prolong the circulation time, escape the endolysosomal compartments, and release the gene drugs in the cytoplasm in a sustained manner. Specifically, Shi *et al*. constructed a unique lipid-polymer NPs platform with PLGA and DSPE-PEG to encapsulate the calcium-activated kinase (CaMKII) siRNA/ C_0_-C_14_ complex. The cationic G_0_-C_14_ effectively absorbed siRNA and allowed it to escape from endosome. The siRNA/C_0_-C_14_ complex was coated by PLGA, and thus avoided serum nuclease degradation of siRNA. DSPE-PEG prolonged the circulating lifetime and protected the siRNA from rapid clearance. Meanwhile, A atherosclerotic macrophage targeting peptide (S_2_P) was modified onto the surface of NPs to promote the specific binding to macrophage stabilin-2 receptors. CaMKII in plaque macrophages of advanced mouse and humans is activated, which promotes the plaque necrosis through the inhibition of the efferocytosis receptor MerTK. By silencing CaMKII gene in lesional macrophages, the siCamk2g NPs increased macrophages MerTK expression, improved efferocytosis function, reduced the necrotic core area, and promoted the stability of the plaques (Figure 4B-4E) 89. Furthermore, Xiao *et al*. developed a near-infrared-II (NIR-II) organic aggregation-induced emission platform based on thienothiadiazole. Composed of PEG-modified NIR-II fluorescent dyes, PLGA, and G_0_–C_14_, this platform encapsulates siRNA targeting the CaMKII gene. It effectively targets CaMKIIγ in macrophages, enabling dynamic imaging of atherosclerosis and image-guided gene therapy (Table 1) 90.

#### 4.2.2. HA based nanomaterials

HA is a naturally occurring linear biomacromolecule-polysaccharide that is a critical component of the extracellular matrix. It is biocompatible, non-toxic, non-immunogenic, and non-inflammatory 91. Due to its specific binding ability to CD44 and stabilin-2, which are richly expressed in activated macrophages, endothelial cells, and smooth muscle cells in atherosclerotic plaques, the biomaterials composed of HA have been extensively studied for the targeted delivery of atherosclerotic theranostic agents 92, 93. Intriguingly, Kluza *et al*. found that the interaction between atherosclerotic macrophages and HA NPs was significantly associated with disease stage. They observed that significantly more HA NPs were phagocytized by macrophages in the early disease stage of ApoE^-/-^ mice than those in advanced stage. Based on these results, they speculated that it might be due to the macrophages in early atherosclerotic lesions with strong phagocytic activity. However, along with the internalization of lipids, and undergoing cell apoptosis and necrosis, the phagocytosis of macrophages in the advanced stage for HA NPs was reduced. In addition, they have found that the atherosclerotic macrophages internalized 6 and 40 times amount of HA NPs compared to those macrophages in the spleen and bone marrow of ApoE^-/-^ mice, respectively (Figure 5A-5D). This result confirmed the excellent macrophage-targeting ability of HA NPs 94. In a subsequent study, the same group revealed the true underlying driving mechanism behind the significant differences in the amount of HA NPs phagocytized by macrophages at different disease stages. The results indicated that, rather than the activity of macrophage, endothelial barrier changed as the atherosclerotic disease progression. Vascular endothelial cells showed better continuity and endothelial normalization in late plaques than that in early plaques. In addition, collagen levels and smooth muscle cells increased strikingly in the late atherosclerotic plaques, which may improve the stabilization of endothelium. In order to be phagocytosed by macrophages, HA NPs should first cross endothelial junctions and then distribute in the endothelial extracellular matrix. Thus, these observations explained why HA NPs in advanced atherosclerotic macrophages were less than that in early macrophages 95.

Based on the targeting ability of HA to macrophages, HA has been widely used as a drug delivery system to deliver small anti-inflammatory molecules to relieve inflammation in AS, or to repolarize macrophages into an anti-inflammatory phenotype by delivering anti-inflammatory factors 96, 97. In particular, HA-based drug delivery systems have also been endowed with trigger responsiveness, such as pH-responsiveness or ROS-responsiveness for controlled drug release or targeted combination therapy of AS 98.

Besides, in view of the high availability of hyaluronidase (HAase) in the plaque extracellular matrix, HA has been used as a “sheddable” corona to protect the nanomaterials from the recognition of reticuloendothelial system (RES), and thus prolonging the blood circulation time, while exposing the binding sites to target cells in plaques. As shown in Figure 5E-5F, Liu and coworkers developed a cell-penetrating peptides (CPPs)/siRNA nanoparticles coated with HA to deliver Lox-1 specific siRNA to atherosclerotic macrophages and inhibit lipid accumulation. The “sheddable” HA corona promoted the location of nanoparticles into the leaky endothelium where CD44 receptors were overexpressed and was further degraded by HAase within the plaque microenvironment, and thus exposing CPP nanocomplexes and eventually entering macrophages. Through the mechanical study, the authors revealed that compared with low molecular weight HA, high molecular weight HA modified nanoparticles with high coating density were more readily to be uptaken by macrophages 99.

#### 4.2.3. Lipid based nanomaterials

HDL is a type of endogenous lipidic nanoparticle composed of phospholipid and apolipoprotein A-i (apoA-i), with the size of 7 ~ 13nm 100. There are two distinct structures of HDL, including discoidal HDL and spherical HDL 101, 102. The discoidal HDL consists of a phospholipid bilayer surrounded by two apo A-i molecules, and the spherical HDL shows a hydrophobic lipid core circumscribed with a phospholipid monolayer with apo A-i embedded 36, 103. Numerous studies have indicated that HDL has anti-atherosclerotic effects, as they can transport cholesterol from lipid-loaded plaque macrophages to the liver for elimination via reverse cholesterol transport 104, 105. In addition, the anti-AS effect of HDL also benefits from its endothelial cell protective function, antioxidant and anti-inflammatory actions 106, 107.

Apart from the potential as antiatherosclerosis therapeutics, HDL nanoparticles have been utilized as nanocarriers for the target delivery of imaging and therapeutic agents to the atherosclerotic macrophages. On one hand, as the most important protein component of HDL, apo A-i shows high affinity for the scavenger receptor SR-B1, which is abundantly expressed on the surface of macrophages. On the other hand, apo A-i with excellent biocompatibility and long circulation half-life can evade the recognition by MPS 103. Given these advantageous functions, HDL nanoparticles are widely used in AS management. On the basis of the advantages of HDL, rHDL prepared from human plasma apoA-1 mixed with a phospholipid films was exploited for the treatment of AS 108, 109. However, the process of extracting apoA-i from human plasma is time-consuming, so synthetic rHDL-like NPs made by assembling apoA-i mimics or bacteria-generated apoA-i with phospholipids are expected to be with similar atheroprotection and drug delivery effects to those of native HDL 110. For instance, Henrich *et al*. reported a straightforward method for the synthesis of HDL mimics by employing lipid-conjugated organic core scaffolds, without the significant time-consuming and expensive enzyme maturation steps. The shape, size, composition, protein secondary structure, and surface chemistry of the prepared HDL-like nanoparticles were consistent with those of mature human HDL. Moreover, the HDL mimics had similar biological activity with mature human HDL. They can significantly promote the cholesterol efflux from macrophages, facilitate the cholesterol transportation to hepatocytes, and inhibit inflammation (Figure 6A-6D) 111. Until now, more advanced rHDL NPs are designed to alleviate the atherosclerotic burden by targeting ligands modification, and loaded with a variety of therapies ranging from specific small molecule inhibitor to RNA 112, 113. What should be noted is that Mulder and coworker showed that the composition of phospholipid, ratio of phospholipid to apo A-1, morphology, and size can affect the *in vivo* performance of rHDL nanoparticles. They created a combinatorial library of 17 rHDL NPs with distinct physiochemical properties via the modulation of composition and synthesis process, and found that there are about 6-fold differences in facilitating cholesterol efflux from macrophages, 10-fold differences in blood half-life, 3.4 times differences in relative aortic to liver accumulation, and 3.8 times differences in relative aortic to spleen macrophage accumulation (Figure 6E). From this library screening, the POPC-dominated rHDL NPs were identified the most favorable compositions, and approximately 30 nm in size and spherical morphology showed the optimal potential to achieve the specific atherosclerotic macrophage-targeted drug delivery 114. The same group also systematically compared the biological behavior of three clinically applicable nanomaterials loaded with the HMG-CoA reductase inhibitor simvastatin, namely high-density lipoprotein ([S]-HDL), liposomes ([S]-LIP), and polymer micelles ([S]-PM) by using quantitative techniques, including positron emission tomography, gamma counting and flow cytometry. The results indicated that [S]-PM and [S]-LIP had long blood circulation time, but the accumulation of three nanomaterials in atherosclerotic plaques showed similar levels. In addition, plaque macrophages displayed the higher uptake of [S]-HDL and [S]-PM than [S]-LIP, while Ly6Chigh monocytes showed the highest uptake of [S]-PM. Among them, [S]-PM possessed the optimal therapeutic function in diminishing macrophage burden in advanced AS 115.

Recent advances in the application of lipid nanoparticles for drug delivery have been highly promising, with several formulations successfully gaining regulatory approval for various pathological conditions. One clinical trial employed liposomal nanoparticles encapsulating prednisolone phosphate. Analysis of plaque macrophages from patients with iliofemoral atherosclerosis scheduled for endarterectomy showed that the majority of plaque macrophages internalized the nanoparticles following intravenous infusion. However, in a broader cohort of cardiovascular disease patients, positron emission tomography (PET) imaging assessment revealed no significant effect of these nanoparticles on arterial wall permeability or inflammatory status. Other researchers have developed a lipid nanoemulsion coating (LDE) that resembles LDL. Upon contact with plasma, this coating acquires apolipoprotein E, enabling its uptake by inflammatory plaque macrophages expressing LDL receptors. Preclinical testing in rabbits demonstrated that LDE-methotrexate nanoparticles, formulated using a lipophilic methotrexate derivative, reduced plaque area by 65%.

#### 4.2.4. Cyclodextrin (CD) based nanomaterials

CD is a kind of cyclic oligosaccharide with unique hydrophobic inner surface, which displays good biocompatibility, biodegradability and low immunogenicity 116. There are three main types of CDs, including *α*, *β* and *γ*-CDs. Benefited from their cost-effective, ease of chemical modification, and ability to form inclusion complexes with various organic and inorganic lipophilic molecules, CDs exhibit distinctive advantages in drug delivery field. For the treatment of AS, β-CD is one of the most widely used cyclodextrins. They can readily form stable inclusion complex with cholesterol and regulate cholesterol metabolism 117. It is reported that cyclic oligosaccharide 2-hydroxypropylb-cyclodextrin can solubilize and entrap cholesterol crystals, and reduce atherosclerotic plaque size in ApoE^-/-^ mice with cholesterol-rich diet. Mechanical studies showed that CD facilitated LXR-mediated transcriptional reprogramming, and thus enhancing reverse cholesterol transport and initiating anti-inflammatory effect 118. However, one of the limitations of free CD in the treatment of AS is the high administration dose required, which probably bring adverse effects. Therefore, the researchers have focused on developing CD based nanomaterials 119, 120. Apart from improving cholesterol efflux, CD NPs have exerted anti-inflammatory function by inhibiting the migration of inflammatory cells and subsequent pro-inflammatory events. Zhang and colleagues constructed a luminol-conjugated *β*-CD (LCD) nanoparticles which can effectively suppress neutrophil-mediated recruitment of inflammatory macrophages and prevent the production of inflammatory response and oxidative stress, thus showing ideal efficacy in the treatment of inflammation-driven diseases, including AS (Figure 7A) 121. On the basis of the fact that *β*-CD is able to host small hydrophobic molecules in hydrophobic cavities, *β*-CD mediated host-guest interactions can be to deliver various therapeutics for AS therapy. A *β*-CD derivative modified macrophage (CD-MP) was constructed by Wang *et al*. as a drug transporter. By a host-guest interaction between *β*-CD and adamantane (ADA), ADA decorated quercetin (QT) encapsulated liposome (QT-NP) was further conjugated to CD-MP to form macrophage-liposome conjugate. Due to the recruitment of macrophages during the progression of AS, macrophages carrying QT-NP "hand-in-hand" anchored in aortic plaques, significantly improving the accumulation of quercetin in the atherosclerotic sites 122. More importantly, the molecules hosted by *β*-CD hydrophobic cavities can be switched competitively according to their affinity with *β*-CD. Based on this, Park *et al*. report a cargo-conversion nanoparticle CSNP, which was consisted of a core formed by the inclusion complex of *β*-CD and simvastatin (statin), and a phospholipid shell. Since cholesterol has higher affinity to *β*-CD than statin, CSNP scavenged cholesterol and released anti-inflammatory statin under the cholesterol-enriched microenvironment through cargo switching. Indeed, in a mouse model of AS, CSNP obviously reduced plaque cholesterol and macrophage levels through synergistic treatment of *β*-CD and statin, thus effectively preventing the occurrence of atherosclerotic plaques and regressing the established plaque (Figure 7B-7E) 123. CD based nanomedicines also take advantage of various factors in the atherosclerotic plaque microenvironment, such as pH, ROS, and enzymes, to achieve trigger-response to improve specificity and treatment outcome of AS 124, 125. For example, Zhang *et al*. constructed an integrin-targeting and pH-responsive nanoparticle (AAM NP) based on acetalated α-CD, cationic polyethyleneimine (PEI), and lipid to deliver anti-miR33 for precise therapy of AS. miR-33 can inhibit cholesterol efflux and HDL biosynthesis during the progression of AS by repressing the expression of ABCA1 and ABCG1. Through lesion-specific delivery and precise regulation of anti-miR33, AAM NP notably promoted the macrophage cholesterol efflux, polarized the macrophages towards anti-inflammatory M2 phenotype, and modulated regulatory T cell differentiation. Consequently, AAM NP treatment effectively de-escalated plaque burden in apolipoprotein E-deficient mice (Figure 7F) 126.

#### 4.2.5. Carbon-based nanomaterials

Since the unique physicochemical properties, carbon-based nanomaterials have shown promising application in various fields, such as drug delivery, tissue engineering, and bioimaging technology. In particular, graphene oxide (GO) nanosheets and carbon nanotubes (especially single-walled carbon nanotubes, SWNTs) have been demonstrated to be effective photothermal absorbers for the AS management via photothermal therapy 127. They are both based on a specific sp^2^ carbon morphology and can transform biologically transparent near-infrared radiation (NIR) at 700-1100 nm to heat. By photothermal conversion, GO nanosheets or SWNTs can induce thermal destruction of macrophages, which shows potential benefit for AS treatment. To enhance their targetability, the surfaces of SWNTs have also been modified with ligands to specifically target macrophages, such as phenoxylated dextran. Phenoxylated dextran modified SWNTs can selectively accumulate in inflammatory macrophages by scavenger-receptor binding, avoiding unnecessary damage to normal cells (Figure 8A-8B) 128. Similarly, a mannosylated reduced GO was prepared to enhance the selection toward mannose receptor over-expressed macrophage for targeted therapy of AS (Figure 8C) 129.

Apart from that, carbon-based nanomaterials show ultrahigh drug loading capacity and favorable non-immunogenicity 130. The backbone of them can be readily modified. So carbon-based nanomaterials are also widely applied to deliver a range of therapeutics. Using these characteristics, Leeper *et al*. developed pro-efferocytic SWNTs (SWNT-SHP1i) for macrophage-specific therapy in AS. SWNT-SHP1i was composed of the PEG functionalized SWNTs as backbone and loaded with fluorescent probe Cy5.5 and small molecule inhibitor of CD47 downstream effectors Src homologous 2 domain-containing phosphatase-1 (SHP-1). The binding of CD47 to signal regulatory protein-*α* (SiRP*α*) on macrophages activates SHP-1, which mediated intracellular cascade signaling pathways. This signaling cascade inhibited the phagocytosis of macrophage and promoted plaque expansion. SWNT-SHP1i effectively interrupted CD47-SIRP*α* signaling in macrophages and enhanced the efferocytosis activity of macrophages. The concentration of SWNT-Cy5.5 in plaque monocytes and macrophages was 70% and 60%, respectively. SWNT-Cy5.5 may selectively target atherosclerotic macrophages in two ways. On the one hand, SWNT-Cy5.5 passively targeted macrophages in the lesions via extravasation due to their nanoscale size. On the other hand, SWNT- Cy5.5 was first taken up by circulating monocytes and then arrive at plaque to be transformed into macrophages during atherogenesis. In addition, the single-cell RNA sequencing of macrophages isolated from the aortas of SWNT-SHP1i treated mice showed that SWNT-SHP1i downregulated pro-inflammatory genes but upregulated anti-inflammatory relevant genes in macrophages. Also, the macrophages in SWNT-SHP1i treated group were enriched with the genes associated with phagocytosis and antigen presentation. Pathway analyses also indicated that SWNTs-SHP1i induced the down-regulation of genes associated with chemokine-mediated signal transduction and chemotaxis of monocytes. In the same year, the same group also applied SWNTs to selectively deliver tyrosine phosphatase inhibitor 1 (TPI) to macrophages, which stimulated the efferocytosis of macrophage to clear apoptotic cell debris by suppressing CD47-SIRP*α* signaling, and thus reducing atherosclerotic disease 131.

Although these exciting findings, some earlier studies have reported that carbon nanotubes damaged the endothelium, promoted monocyte adhesion and calcification, and thus contributed to the development of AS 132, 133. It is urgent and important for carbon-based materials to further systematically investigate their biological effects in future.

#### 4.2.6. Biomimetic nanomaterials

Biomimetic nanomaterials are an innovative kind of materials that combine the advantages of natural and synthetic materials. They avoid the loaded drugs being cleared quickly from the blood circulation and extending circulation time. They also show excellent targetability and efficient immune escape ability 134, 135. These favorable characteristics drive the wide application of biomimetic nanomaterials in the treatment of AS.

On the one hand, on the basis of the phagocytosis effect of macrophages on apoptotic bodies released by apoptotic cells, apoptotic body-biomimic nanomaterials are constructed to target inflammatory macrophages for the treatment of AS. Specifically, it has been reported that the recognition and phagocytosis of macrophages to apoptotic bodies *in vivo* are based on the surface exposure of phospholipid serine (PtdSer) that triggers the "eat-me" signals of macrophages. According to this, Zhang and coworkers prepared an apoptotic body biomimic liposome (AP-Lipo) by decorating PtdSer on the surfaces of liposomes to selectively deliver PPARγ agonist (pioglitazone) into atherosclerotic macrophages. Compared with undecorated liposomes, such biomimic AP-Lipo more effectively targeted proinflammatory macrophages and up-regulated anti-inflammatory cytokines to inhibit inflammation, thus suppressing the progression of AS 136.

On the other hand, in light of the "homing" of macrophages into atherosclerotic lesions, biomimetic nanoparticles coated with macrophage membranes have been shown to be an effective drug delivery system for AS therapy 137. In this case, a macrophage membrane coated PLGA nanoparticle was prepared to deliver RAP for targeted anti-AS applications. Such macrophage membrane camouflaged NPs can significantly accumulate in atherosclerotic plaques, reduce the secretion of inflammatory cytokines in macrophages, and effectively lower atherosclerotic lesion areas 138. To realize intelligent delivery, the ROS-responsive macrophage membrane coated NPs were prepared for controlled release of payloads at the atherosclerotic lesions 139. As shown in Figure 9A-9C, ROS-responsive NPs (MM-AT-NPs) were constructed by amphiphilic phenylboronic acid pinacol ester grafted chitosan oligosaccharide, then loaded with atorvastatin and coated with macrophage membrane. After arrived at target inflammatory tissue, MM-AT-NPs responded to local excess ROS, inducing the effective pharmacotherapy. Moreover, the surface of MM-AT-NPs contained main membrane antigens (e.g., CD36, CCR2, TNFR2) that sequester proinflammatory cytokines to inhibit local inflammation. Through the synergistic action of inflammatory cytokines sequestration and drug delivery, this biomimetic NPs significantly improved the therapeutic effect of AS. Notably, live macrophages were also applied to replace macrophage membrane to prepare AT-NP/MAs in this study. Compared to MM-AT-NPs, AT-NP/MAs showed a higher accumulation in atherosclerotic tissues due to the strong targeting ability of living macrophages. However, unlike MM-AT-NPs, AT-NP/MAs may bring negative effects on the AS therapy as the live macrophages are readily activated by inflammatory cytokines to produce more proinflammatory cytokines 140. To avoid the expensive cell membrane collection, the exosomes secreted from macrophage are also employed to construct biomimetic nanoparticles 141. Principally, Xie *et al*. used the molecularly engineered M2 macrophage-derived exosomes to electroporate with hexyl 5-aminolevulinate hydrochloride (HAL) to generate HAL@M2 Exo. Engineered exosomes derived from M2 macrophages displayed intrinsic inflammatory tropism and anti-inflammatory ability. After systematic administration, HAL@M2 Exo Exo effectively targeted atherosclerotic lesions via surface-bonded chemokine receptors and released anti-inflammatory cytokines to relieve inflammation in atherosclerotic lesions. In addition, the encapsulated HAL was catalyzed by a series of enzymes to synthesize heme and further generate anti-inflammatory carbon monoxide and bilirubin, which amplified the anti-inflammatory effects and ultimately boosted the remission of AS (Figure 9D-9F) 142. What is notable is that the exosomes may contain mixtures of multiple components. It will be better if the variations from batch-to-batch could be addressed.

## 5. Nanomaterials targeting atherosclerotic macrophages for AS diagnosis

Imaging is essential for diagnosis and management of patients with AS. Macrophages play an important role in the development and acute complications of AS, which provides convincing biomarker for identifying subclinical inflammatory lesions, predicting future risk, and aiding to assess novel treatments. Among various imaging modalities, CT, magnetic resonance imaging (MRI), positron emission tomography (PET), ultrasound imaging, photoacoustic imaging (PAI), and fluorescence imaging (FI) are the most common imaging modalities employed to identify and assess plaque progression in the arterial system. Specifically, CT scans apply X-ray measurements to create cross-sectional, tomographic "slices" of an interest area at different angles with high spatial resolution and no tissue penetration limitations. Calcium is most readily to identify in plaque imaging 143, 144. However, the signal attenuation is inevitable due to the plaque heterogeneity, which impacts the specific imaging of atherosclerotic lesions. Currently, clinical CT scanning technology exhibits high specificity, enabling the detection of atherosclerotic burden, stenosis degree, and plaque calcification with good reproducibility. However, it suffers from low spatial resolution in distinguishing soft tissues and carries risks associated with radiation exposure and iodinated contrast agents. In contrast to CT, MRI does not involve X-rays. The protons in hydrogen atoms abundant in our bodies align in the direction of the magnetic field. After being affected by a short radio-frequency pulse, these protons realign with the magnetic field to emit a detectable signal. Observations and images can be made based on the comparison of MR signal intensity between different tissues caused by the number of protons. With the aid of paramagnetic species, contrast imaging can be obtained 145, 146. Clinical studies have demonstrated that MRI offers advantages such as high spatial resolution and excellent tissue penetration, providing richer morphological information (including necrotic core and hemorrhage). However, it can only detect lesions without elucidating pathological mechanism and is prone to artifacts caused by small vessel motion. In addition, metal implants bring a risk during MRI imaging. MRI also needs high cost and long operation time 147. PET is another common imaging technique based on γ-ray. PET scanning images specific tissues by intravenous injection of radioisotope-labeled ligands or tracers with high sensitivity and excellent tissue penetration 148. However, the anatomical resolution of PET imaging is poor and needs to be confirmed by combination with CT or MRI 149. Ultrasound as a low-cost and fast imaging approach can be used for real-time observation. However, it has a low resolution and is limited to image specific organs or bones 150. Currently, clinical intravascular ultrasound technology offers the advantage of strong penetration depth and can differentiate luminal dimensions and plaque burden. However, it is invasive and operator-dependent. PAI, another ultrasound-based imaging technique, has also been used. However, due to limitations such as tissue penetration of light, PAI and fluorescence imaging technology have not been widely applied in the medical industry to identify plaques as other mentioned cardiovascular disease imaging approaches 151.

Currently, commonly used clinical diagnostic techniques for atherosclerosis primarily provide information at the plaque structural and anatomical levels. However, these technologies remain insufficient in distinguishing between vulnerable and stable plaques, making it difficult to accurately identify vulnerable plaques in their early stages. Theoretically, this limitation can be attributed to the fact that existing clinical imaging methods fundamentally rely on the detection of structural abnormalities. Molecular imaging is increasingly playing a significant role in the early diagnosis of diseases. Given the characteristic pathophysiological changes that occur during the formation of vulnerable plaques, probes targeting specific biological features are expected to provide additional insights into suspicious plaques. The accumulation of a large number of macrophages within plaques is a critical hallmark associated with fibrous cap thinning. The rational design of nanomaterial-based imaging contrast agents that can specifically target macrophages within plaques enables the visualization of atherosclerotic plaques prone to rupture or erosion. This approach not only offers key insights into plaque biology but also facilitates the quantification of atherosclerotic burden and the evaluation of therapeutic efficacy at the molecular, cellular, and functional levels.

The emergence of nanomaterials holds great promise for breaking the limitations of these common imaging modalities. Nanomaterial assisted imaging is a method to use exogenous contrast agents to image the lesions during the development and progression of AS. First, nanoparticles can be employed to image the site of interest with conventional devices 152. For example, the metallic elements are synthesized into superparamagnetic or paramagnetic nanoparticles. Their size, shape and electronic charge can be effectively controlled, which will directly affect the non-specific absorption and biological distribution of nanomaterials. Secondly, the methods of material synthesis and surface functionalization have been greatly improved in recent years. The surfaces of nanoparticles can be readily modified 153. For instance, by coating the nanoparticles with specific peptides, the precise targeting to macrophages at the site of atherosclerotic disease can be achieved. Third, the absence of radioactivity and limited toxicity further encourage the employment of nanotechnology. Taken together, the unique physical and biological advantages of nanoparticles make them an ideal candidate for imaging of cardiovascular disease (Table 2).

### 5.1. Iron oxide nanoparticles

Iron oxide nanoparticles is a kind of metallic crystal materials with intrinsic magnetic property, composed of magnetite (Fe_3_O_4_) and magnetite (Fe_2_O_3_). The shape and size of synthetic iron oxide NPs can be carefully controlled for various applications, from spherical, cluster, and cubic to linear 154. Iron oxide NPs have been extensively studied as T_2_ contrast agents in MRI imaging. They effectively shorten the relaxation time for T_2_-weighted imaging and reduce the signal intensity on T_2_-weighted imaging 155, 156. Particularly, iron oxide NPs with favorable biocompatibility and degradation mechanisms, are easy to be phagocytosed by plaque macrophages, induce signal loss in T_2_-weighted imaging, and thus improving the image contrast of arterial wall plaques 157. Moreover, modification of targeting moieties on the surfaces of superparamagnetic iron oxide nanoparticle (SPION), such as dextran sulfate, human ferritin protein cage, PP1 peptide, osteopontin (OPN), or annexin V90, can specifically target SPION to focal macrophages to increase SPIONs accumulation in vulnerable aortic or carotid plaques, show good MRI negative contrast enhancement for arterial thrombosis and improve the diagnosis of early AS and vulnerable plaque 158, 159. In clinical practice, SPION-enhanced MRI technology has been utilized to assess macrophage-associated inflammatory burden and monitor the efficacy of lipid-lowering therapy.

### 5.2. Gold (Au) NPs

Gold (Au) NPs have also been used to improve the diagnostic process of AS. Au NPs provide excellent surface properties, mature synthesis processes and good biocompatibility. On the basis of its high atomic number and X-ray absorption coefficient, Au NPs have been explored as contrast agents for X-ray CT imaging in identification of atherosclerotic plaques 160. Studies have shown that Au NPs have better imaging effect than iodine contrast agent under the same conditions 161. In addition, because of the surface plasmon resonance (SPR) effect of Au NPs, they as attractive probes have been used for surface-enhanced Raman imaging and fluorescence imaging. Notably, the Au NPs with different configurations and sizes possess different SPR absorption and scattering spectrum 162. Among all, the longitudinal absorption peak of Au nanorods can be regulated from the visible region to near infrared region, and thus Au nanorods show great potential in the diagnosis and treatment of AS. By modifying certain ligands on gold nanorods, gold nanorods can specifically interact with plaque macrophages for bioimaging. Compared with fluorescent molecules and semiconductor quantum dots, Au NPs exhibit unique superiority, such as high light intensity, good stability, and easy preparation 163. For example, Li *et al*. constructed a dual-modality imaging probe (PP1-Au@GSH@Gd NCs) by covalently linking peptidic PP1 to gold nanoclusters integrated with gadolinium. PP1-Au@GSH@Gd NCs can specifically bind to SR-AI ligand on activated macrophages and showed preferential macrophage targeting effect. In established ApoE^-/-^ mice models, PP1-Au@GSH@Gd NCs enabled comprehensive and precise diagnosis of atherosclerosis with superb spatial resolution and detectivity 164.

### 5.3. Upconversion nanoparticles (UCNPs)

Upconversion nanoparticles (UCNPs) as optical imaging tools exhibit sharp emission peaks, tunable multicolor emission, improved quantum yield, and negligible light scattering. On the basis of these promising advantages, UCNPs have been applied to explore the specific characteristics of plaque macrophages. As shown in Figure 10A, Tang *et al*. prepared a luminescence resonance energy transfer (LRET)-based upconversion luminescent nanoprobe to dynamic monitor the activation of CD36 and direct image the binging of CD36 and oxLDL on the macrophage cell surfaces. The luminescent nanoprobe donor was composed of NaYF4:Yb,Er coated with silicon dioxide (SiO_2_) layer and modified with a CD36 antibody (UCNFP@SiO_2_-CD36), which can specifically bind to CD36 antigens on RAW 264.7 macrophage cell membrane. DiI-labeled oxLDL (DiI-oxLDL) acted as an energy acceptor. During the formation process of macrophage foam cell, DiI-oxLDL binded to CD36, which made DiI-oxLDL and the nanoprobe close, and thus generated fluorescence signals via LRET under the continuous wave excitation at 980 nm. The excess DiI-oxLDL in the cytoplasm was far away from the nanoprobe, so the fluorescence was not emitted. In addition, nanoprobe exhibited high sensitivity and enabled effective identification of signaling changes correlated with the distinct features of CD36 165. However, this system has only been used to elucidate AS signals at the cellular level, and not been applied for *in vivo* imaging. Cao *et al*. constructed anti-MARCO upconversion nanoprobes by modifying the polyclonal MARCO antibody to the surface of NaGdF4:Yb,Er@NaGdF4 upconversion nanoparticles for imaging the polarization of M1 macrophages in atherosclerotic plaques. Based on the fact that macrophage receptors with MARCO are obviously upregulated on the surfaces of M1 phenotype macrophages, anti-MARCO UCNPs probes specifically targeted M1 macrophages or ox-LDL induced foamy macrophages, and showed great potential for elucidating the biological action of macrophages in vulnerable plaques non-invasively 166.

### 5.4. Quantum dots (QDs)

Quantum dots (QDs) are nanoscale fluorescent semiconductor crystals whose emission spectra can be precisely tuned by changing the size of the QDs. They have wide excitation band and narrow emission band, remarkable optical stability, high quantum yield and long fluorescence lifetime, which make them act as superior substitutes for organic dyes in disease diagnosis 167. In particular, the near infrared quantum dots (NIR QDs) with excitation and emission light window at 700-1200nm can effectively avoid the interference from tissue absorbance and scattering, and thus provide an ideal choice for the construction of fluorescence probes for AS imaging with high detection sensitivity 168. To reduce the wide systemic biodistribution, QDs were decorated with specific biological molecules or encapsulated with various nanocarrier systems to improve their accumulation and retention in the targets of AS. For example, Cui *et al*. constructed multifunctional Simian virus 40 (SV40) nanoparticles (SV40 VLPs) encapsulated with near-infrared QDs and bearing three targeting peptides for noninvasive fluorescence imaging of atherosclerotic plaques (Figure 10B). These QDs-based SV40 VNPs showed the great potential to image and differentiate early, developmental, and late stages of AS in living ApoE^-/-^ mice by integrating the certain targeted peptides that target atherosclerotic markers vascular cell adhesion molecule-1, macrophages, and fibrin, respectively. Moreover, SV40 VNPs could also be used to targeted deliver the therapeutic drugs Hirulog and acted as a good “theranostic” platforms for the detection and treatment of AS with prominent optical properties 169.

### 5.5. Semiconducting polymer nanoparticles

The π-conjugated semiconducting polymer nanoparticles show the advantages of adjustable optical properties, high extinction coefficient, controlled size, fantastic photostability and biocompatibility 170. As photoacoustic contrast agents, they display better PA performance than other types of contrast agents. Therefore, they become a "hot spot" in the field of biomedicine in recent years, and have been applied in the diagnosis and treatment of AS. Dong *et al*. confirmed the feasibility and potential of using semiconductor polymers-based PA imaging techniques with high sensitivity and excellent imaging resolution to evaluate atherosclerotic inflammation *in vivo* for the first time. They synthesized an anti-CD36 modified semiconductor polymer nanoparticle (PBD-CD36) with an absorption wavelength in the NIR-II region (1000-1400 nm). PBD-CD36 can label inflammatory macrophages and display atherosclerotic inflammation via PA imaging. In *in vivo* experiments, the larger the proportion of CD36 positive expression area in the corresponding CD36 staining area of carotid artery, the stronger the photoacoustic signal of PBD-CD36 nanoprobe. The smallest proportion of CD36 positive expression area in the intima-media area that could be detected was 0.008 171. Wang and coworkers synthesized a 3, 6-bis (4-methylthiophen-2-yl)-2, 5-bis (2-octyldodecyl) pyrrole [3,4-c] pyrrole-1,4(2H,5H)-dione-based π-conjugated polymer (PMeTPP-MBT) as a photoacoustic probe. On this basis, the targeted theranostic nanoparticle (PA/ASePSD) was constructed by combining astaxanthin with SS-31 peptide and loaded with PMeTPP-MBT for non-invasive photoacoustic imaging and multi-channel synergy therapy of AS. The dextran shell of the nanoparticle endowed it with active targeting to atherosclerotic lesions by binding to highly expressed VCAM-1 and CD44 receptors on macrophages and damaged endothelium. The acid plaque microenvironment and high levels of ROS acting as a smart cascade switch triggered the controlled release of astaxanthin, mitochondrial targeting SS-31 peptide and PMeTPP-MBT. Astaxanthin increased the expression of ABCA1/G1, a vital protein, that controlled cholesterol efflux in foam cells, while SS-31 peptide down-regulated the expression of LOX-1/CD36 and suppressed the uptake of ox-LDL by macrophages. Through anti-inflammation and lipid metabolism regulation, the impressive anti-atherosclerotic capability was achieved. Moreover, the released PMeTPP-MBT endowed this system with non-invasive photoacoustic diagnostic capability. The photoacoustic imaging results were consistent with the results presented by ORO staining. These promising results demonstrated that PA/ASePSD nanoplatform had great clinical potential for accurate diagnosis and effective therapy of early-stage AS (Figure 10C-10D) 172. Fan and colleagues utilized semiconducting conjugated polymers-organic molecules with π-conjugated backbones-to construct a macrophage-targeted theranostic platform capable of delivering si-Olfr2, which integrates NIR-II photoacoustic imaging and siRNA therapeutic approaches. This platform aims to achieve both downregulation of Olfr2 expression in lesional macrophages for atherosclerotic treatment and high-resolution NIR-II photoacoustic imaging for AS diagnosis. Leveraging a reactive oxygen species (ROS)-responsive nanocarrier system, the platform effectively suppresses Olfr2 expression in macrophages within atherosclerotic plaques, thereby inhibiting NLRP3 inflammasome activation and IL-1β secretion, ultimately reducing plaque formation. Concurrently, it enables high-resolution NIR-II photoacoustic imaging of atherosclerotic plaques 173.

### 5.6. Fluorescent molecule-loaded Nanoparticles

Organic fluorescent molecules show attractive advantages such as controlled synthesis, flexible design and easy to use. Many of them have been commercialized. But they also face some intractable problems in biological applications, such as poor optical stability and no targetability 174. Nanomaterials are not only applied as delivery cargos for therapeutics to treat AS, but also can be modified or loaded with near-infrared fluorophore to achieve molecular and cellular imaging of AS. A variety of near-infrared fluorophore incorporated nanomaterials have been prepared to target atherosclerotic plaque macrophages, including polymeric NPs, liposomes, liposomes, peptide-based NPs, protein cage structure and so on 153. These nanomaterials demonstrate a high affinity for phagocytic macrophages. In addition, decoration with target moieties that specifically bind to macrophage surface receptors also enables the nanomaterials show great potential to detect the macrophage burden in atherosclerotic plaques 170. Compared with small molecule fluorescence agents, fluorescence molecule-based nanoprobes not only display good biocompatibility, prolonged blood circulation time and specific plaque targeting, but also can be readily designed as activatable probes that are switched from "OFF" to "ON" to produce effective signals in response to specific stimuli, and thus show great promise for detecting disease information with a high signal-to-noise ratio 175. Ogawa *et al*. synthesized a novel near-infrared fluorescence-activatable switch-on probe Peptide-ICG2, in which the fluorescence of ICG is quenched via conjugating it to a linker peptide with quenching effect. When the peptide was cleaved by the lysosomal enzyme cathepsin B, the fluorescence can be recovered and detected. Because cathepsin B is over-expressed in the lysosomes of macrophages, fluorescence was dominantly turned on in macrophages. To achieve specific imaging of plaque macrophage, a phosphatidylserine (PS)-containing liposome was prepared as the carrier for the delivery of Peptide-ICG2. Since highly expressed PS receptor is one of the main characteristics of macrophages in embolism-vulnerable plaques, this system can effectively accumulate into macrophage infiltrating into the atherosclerotic plaques. Using this approach, they successfully detected pathological plaques in atherosclerotic mice and rabbits 176. Such activatable probe with macrophage targeting showed high signal-to-noise ratio, and greatly improved the detection sensitivity of fluorescence imaging in cardiopathy diagnosis.

### 5.7. MRI Contrast agents-loaded Nanoparticles

Gadolinium (Gd)-based contrast agents can be used to detect atherosclerotic lesions via MRI. These contrast agents reduce T1 relaxation property, shorten the relaxation time for T1-weighted imaging, and thus increasing MR scan signal intensity and improving imaging tissue resolution. However, conventional Gd contrast agents suffer from obvious deficiencies, such as long scan time required, rapid renal clearance, lack of specificity, and toxicity as a result of their non-specific biodistribution. These deficiencies are particularly relevant to their application in AS diagnosis since Gd-based contrast agents arrived at diseased plaques cannot meet the level of required for early detection and anatomic characterization of AS in clinical practice 177. Gd-incorporated nanoparticles have been shown to boost the relaxation properties of Gd-based contrast agents, improve their pharmacokinetic behavior, enhance their efficiency for visualization of atherosclerotic anatomy while reduce systemic toxicity 178. In particular, modification of nanoparticles incorporated with Gd are able to further promote the specific accumulation of Gd-based contrast agents in atherosclerotic sites by targeting macrophage surface receptors, and thus allowing an earlier identification of patients with more accurate response. Macrophage-specific recognition ligands such as anti-CD36 antibody, RGD peptides and hyaluronic acid have been used for the construction of these imaging contrasts 179. Zhou *et al*. prepared a HA-guided cerasomes (HA-CC) and loaded with rosuvastatin (RST) and contrast agent Gd for selective delivery of therapeutic and diagnostic agents to atherosclerotic plaques. The high affinity between the HA and CD44 positive macrophages, vascular endothelial cells and smooth muscle cells in plaques endowed it improved targeting ability to atherosclerotic plaques. Compared with the treatment with free drug, the HA-CC-RST treated plaque regressed significantly. In addition, Gd-loaded HA-CC showed outstanding ability to recognize vulnerable plaques with high contrast *in vivo* viα MRI imaging. In comparation with non-targeted CC-Gd or free Gd agent, HA-CC-Gd with the dose of 0.5 mg/kg enhanced the MRI signal intensity by about 80% and 40% after administration for 6 h (Figure 11A-11B). Thus, HA-CC was expected to achieve the goal of improving simultaneous therapy and imaging of vulnerable plaques 96. Zhang *et al*. developed a dual-functional nanosystem—arginine and gadolinium ion co-doped polydopamine nanoparticles (abbreviated as AGPDAR-146a). The AGPDAR-146a nanoparticles can efficiently load and deliver miR-146a. Through the specific interaction between miR-146a and class A scavenger receptors, they achieve targeted accumulation in inflammatory macrophages, thereby effectively promoting the regression and stabilization of atherosclerotic plaques. Furthermore, these nanoparticles possess T_1_-weighted magnetic resonance imaging capability, enabling real-time visualization of plaque inflammatory progression. This approach addresses the dual needs for diagnostic and therapeutic strategies in atherosclerotic plaque management, offering a valuable diagnostic tool while fulfilling its therapeutic functions (Figure 11C-11D) 180.

### 5.8. Radioactive tracers -conjugated Nanoparticles

Radioactive PET tracers are able to image inflammatory atherosclerotic plaques. ^18^F-fluoro-2-deoxyglucose (^18^F-FDG) and ^18^F-sodium fluoride (^18^F-NaF) are two extensively used radioactive tracers that have been applied to image vascular inflammation or calcification activity to reflect the progression of AS. In particular, ^18^F-FDG can be avidly uptaken by inflamed macrophages, and thus it has been proposed as the gold standard for noninvasive identification of vulnerable atherosclerotic plaques by detecting macrophage burden and vascular inflammation 181. However, using ^18^F-FDG to image plaque macrophages in coronary arteries is particularly challenging since ^18^F-FDG is not specific for macrophages and it can also be highly uptook by cardiomyocytes to participate in the myocardial metabolism, which inevitably causes false positive signals. Moreover, due to the limited spatial resolution (~ 2mm) of PET as well as the small size of atherosclerotic plaques, a high degree of ^18^F-FDG accumulation in lesions is required for the AS characterization. Radioactive tracer-labelled nanoparticles show tremendous promise for solving these problems because they have good pharmacokinetics and are able to visualize macrophages in atherosclerotic plaques specifically and quantitatively. Nahrendorf *et al*. created an ^18^F-modified polyglucose nanoparticle (^18^F-Macroflor) as the PET imaging agent to detect atherosclerotic plaques in cardiovascular tissues. Because of the high affinity of ^18^F-Macroflor to lesional macrophages, it significantly increased the PET signal in infarct regions and atherosclerotic plaques in both mouse and rabbit models. Moreover, since the ^18^F radioisotope decays at a fast rate (T_1/2_ 110 minutes), they shrank the size of Macroflor to below the renal excretion threshold and made them can be excreted rapidly to match the short half-life of ^18^F, which was more conducive for imaging inflamed macrophages in cardiovascular organs. As a result, ^18^F Macroflor successfully identified the changes in macrophage population size with spatial resolution (Figure 11E-11G) 182. On the basis of high avidity between Macrin and inflammatory macrophages, the same group also constructed ^64^Cu-Macrin and confirmed that ^64^Cu-Macrin PET could act as a feasible PET nanotracer to study the spatiotemporal dynamics of macrophages in a variety of pathological conditions, such as atherosclerotic plaque, septic and ischemic myocardial, lung after acute myocardial infarction, and subclinical and clinical pneumonia, to assist disease management 183.

Apart from that, ^89^Zr, ^99m^Tc and ^68^Ga-labelled nanoparticles have also been extensively applied to detect plaque macrophages and access the severity of AS 81, 94, 184.

## 6. Conclusion and Future Perspectives

In general, AS is a persistent, slowly progressing vascular lesion disease and is a complex pathophysiological process involving a variety of cells and factors, implicating multiple blood vessels throughout the body. Recent studies have highlighted important cellular biological atherogenic processes, including the origin and contribution of damaged macrophages. Macrophages play a significant role in the whole process of AS from vascular damage to plaque rupture. The imperative role of macrophages in the incidence and progression of AS provides a theoretical basis for the construction of engineered nanomedicines. In this regard, the delivery of therapies targeting plaque macrophages is superior to systemic delivery in regression of atherosclerotic plaques without compromising host immune defenses. An in-depth understanding of the journey from nanomedicine to macrophage could contribute to the more refined design of nanomedicine to specifically target macrophages with high efficiency. More and more multifunctional nanomaterials have sprung up, such as HA NPs, CD NPs, rHDL NPs and biomimetic NPs, to target plaque macrophages, inhibit macrophage precipitation, remodel macrophage phenotype, restore the fluid-making capacity of macrophages, reduce the inflammatory response, maintain cholesterol homeostasis in macrophages, regulate macrophage apoptosis, and promote macrophage autophagy for AS treatment. Apart from improving the delivery efficiency of carried therapeutics, these nanomaterials also show exceptional ability to specifically interact with macrophage to intervene the atherosclerotic processes. In addition, these nanoparticles can also be tailor-made to combine with other smart functions to fight against atherosclerotic plaque formation. The application of nanomaterials customized for plaque macrophages is not only limited to the treatment of AS, they have also been designed and employed to improve the imaging and diagnosis of AS. The design and construction of an engineered nanoimaging platform enables atherosclerotic lesion imaging with greater sensitivity, higher contrast and deep tissue penetration depth, which is contributed to the early detection, treatment and intervention of atherosclerotic diseases. Nevertheless, current strategies are still in the preliminary research stage, and more effective macrophage-targeted nanotechnologies are expected to be established in the future.

First, the phenotype and functions of macrophages in AS and their interactions with other cells are still needed to further explore. By further revealing the pathogenesis of AS and in-depth understanding the microenvironment of vulnerable plaques, we may find novel potential diagnostic and therapeutic targets, thereby improving the treatment efficiency and realizing more specifical imaging for AS. Recently, atherosclerotic plaque progression based on single-cell sequencing has discovered more significant functional heterogeneity of macrophages in plaques, including activated different phenotypes and foam cell transcriptional signatures. Single-cell sequencing, along with transcriptomic and epigenomic analysis, can reveal cell profiles with higher spatial and temporal resolution. Temporal studies can clarify the dynamic phenotype changes of macrophages. The single-cell analysis will also enable us in-depth understand cell-cell interactions in diseased areas. It can be predicted that, the ever-deeper understanding of the role of macrophages in the pathogenesis of AS will create new advances in the prevention of CVDs. Second, beside discovering novel therapies, the nanoplatforms need to be further optimized. Although many nanomaterials are tailored to target macrophages, the targeting efficiency of nanomaterials is one of the main concerns. Studies have shown that adjusting the size of nanomaterials can alter their interactions with macrophages in vascular diseases. The morphology of nanomaterials, such as spherical, rod-shaped, linear, disk-shaped, etc., also affects their interactions with cells and behaviors such as vascular wall margination. The curvature of spherical nanomaterials limits their contact area with the vascular endothelial surface, reducing their tendency for margination and adhesion. In a plate flow chamber model, rod-shaped particles with high aspect ratios exhibited superior adhesive properties compared to conventional spherical particles. This indicates that rod-shaped nanomaterials are more prominent in terms of targeting efficacy. Additionally, nanomaterials with different morphologies display distinct selective preferences for various inflammatory cell subpopulations. Among three nanostructures with identical surface chemical properties but different structures—micelles, polymersomes, and filomicelles—micelles demonstrated higher specificity for macrophages and dendritic cells in the liver. Furthermore, surface properties are also crucial factors in the design of nanomaterials. Due to the presence of negatively charged sialic acid groups on the macrophage membrane surface, positively charged nanomaterials are more readily endocytosed by macrophages. The CD36 receptor on macrophages can slightly enhance the endocytosis of negatively charged nanomaterials. And the precision and sensitivity of intelligent nanomaterials should be systematically explored to improve imaging and therapeutic efficacy in AS in future studies. In addition, the residence and subsequent metabolism of nanomaterials in the body, as well as the possible immune response that nanomaterials cause, need to be revealed in detail with in-depth research. The sustainability, biodistribution, biosafety, and clearance of various nanomaterials, as well as their stability must be taken into account before the clinical transformation. What's more, although some nanomaterials possess good biocompatibility and show superior biosafety in other diseases treatment and diagnosis, they may not be suitable for anti-AS research. Therefore, we need to comprehensively explore and uncover the action mechanisms of nanomaterials in plaque management. Last, while anti-AS therapy for multiple links of macrophage targets has been demonstrated to be efficacious in some animal research settings, their actual clinical translation has been rather lacking. On the one hand, the complex construction of nanomaterials leads to complicated preparation and quality control processes, poor repeatability from batch to batch, poor storage stability and high costs, all of which hinder large-scale production. On the other hand, there are huge differences in the pathbiology of AS between animals and humans. Despite the positive results in animal studies, these animal models do not completely mimic human physiology and pathology of AS. Therefore, successful experience with macrophage-based therapies does not yet guarantee that it will be as effective in humans. Together, macrophages-targeted nanotechnology-based diagnostic and therapeutic strategies have brought great hope and possibilities for the precise management of AS. Further accumulating studies remain to be conducted.

## Figures and Tables

**Figure 1:**
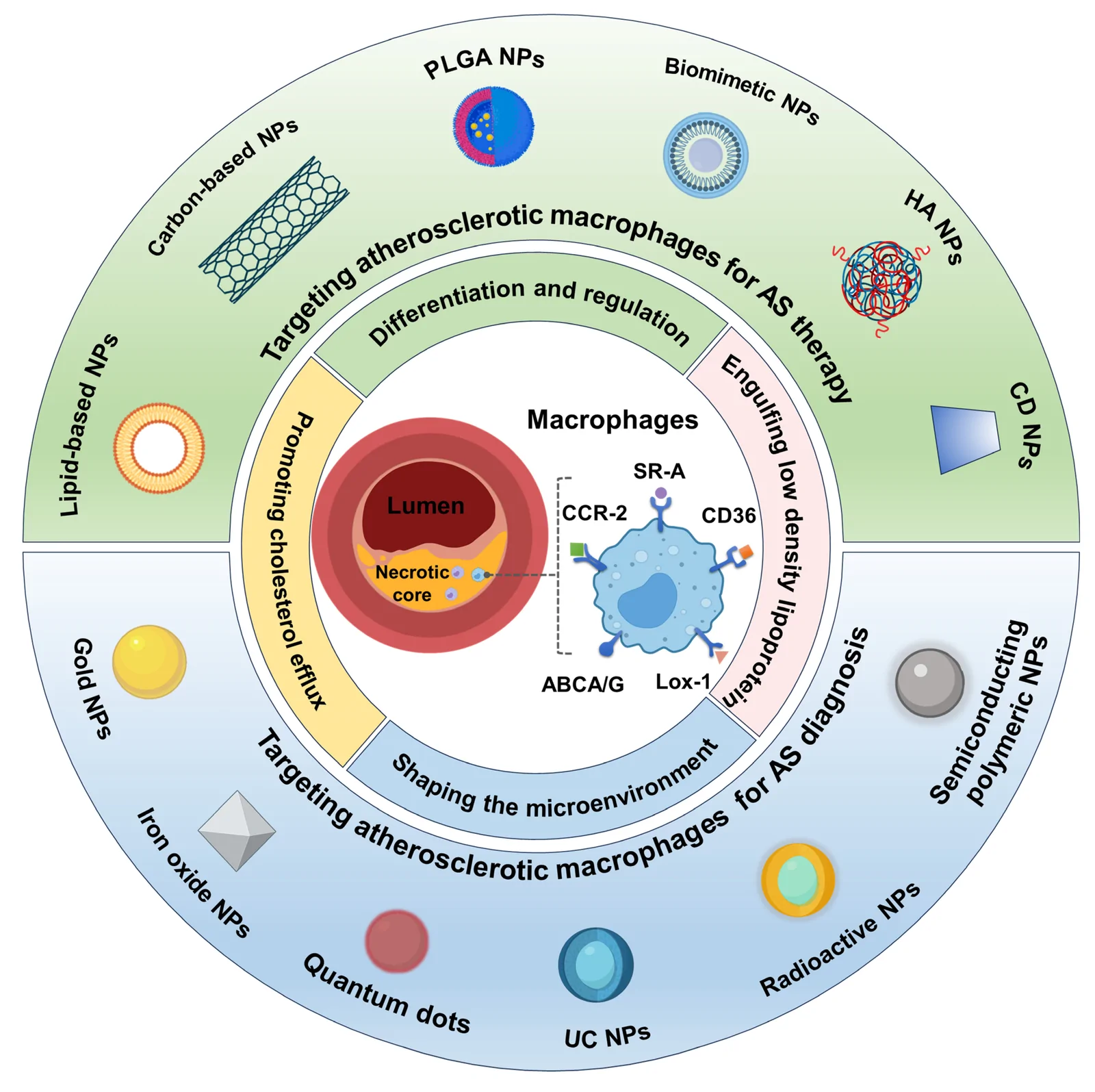
Schematic illustration of macrophage-targeted nanomaterials discussed in this review for the diagnosis and treatment of AS, as well as the characteristics and functions of macrophages in AS.

**Figure 2:**
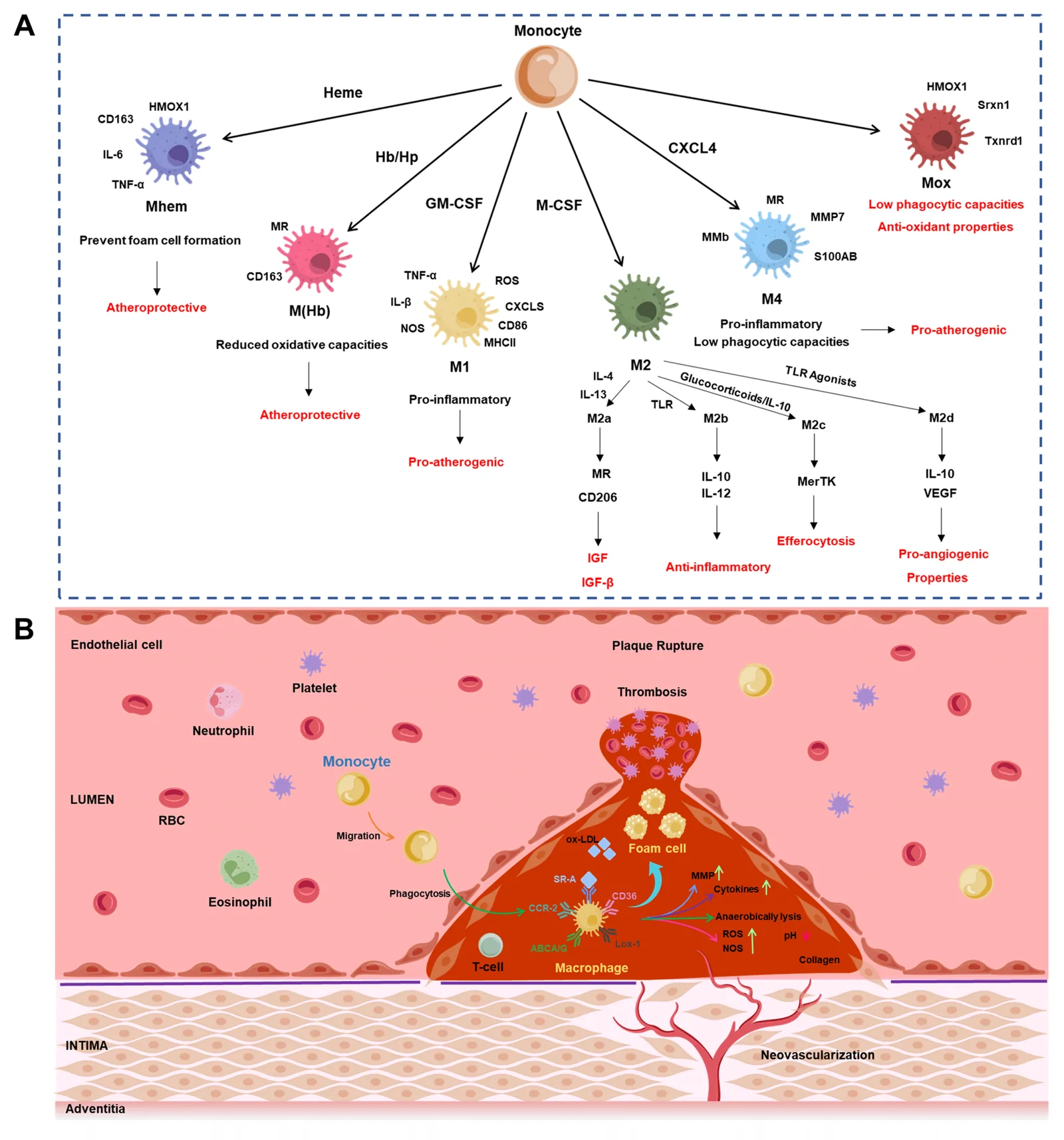
** The polarization and role of macrophages in AS.** (A) Polarization of macrophages. In atherosclerotic lesions, macrophages can differentiate into various phenotypes including M1, M2, M4, M(Hb), Mox, and Mhem. M2 macrophages can be further subdivided into M2a, M2b, M2c, and M2d subtypes. (B) Foam cell formation and the role of macrophages in the development of AS. Macrophages in AS are mainly derived from monocytes. Recruited monocytes differentiate into macrophages and phagocytize oxidized low-density lipoprotein (OX-LDL) to form foam cells. Macrophages express various scavenger receptors (SRs) on their surface, through which they take up lipoproteins. The involved SRs include SR-A, CD36, Lox-1, and others. In addition, macrophages secrete chemokines and inflammatory cytokines to induce an inflammatory microenvironment; shift to anaerobic glycolytic metabolism, thereby reducing the pH in the plaque microenvironment; generate ROS through the NADPH oxidase system; and overproduce proteases that destabilize atherosclerotic plaques and erode the fibrous cap.

**Figure 3:**
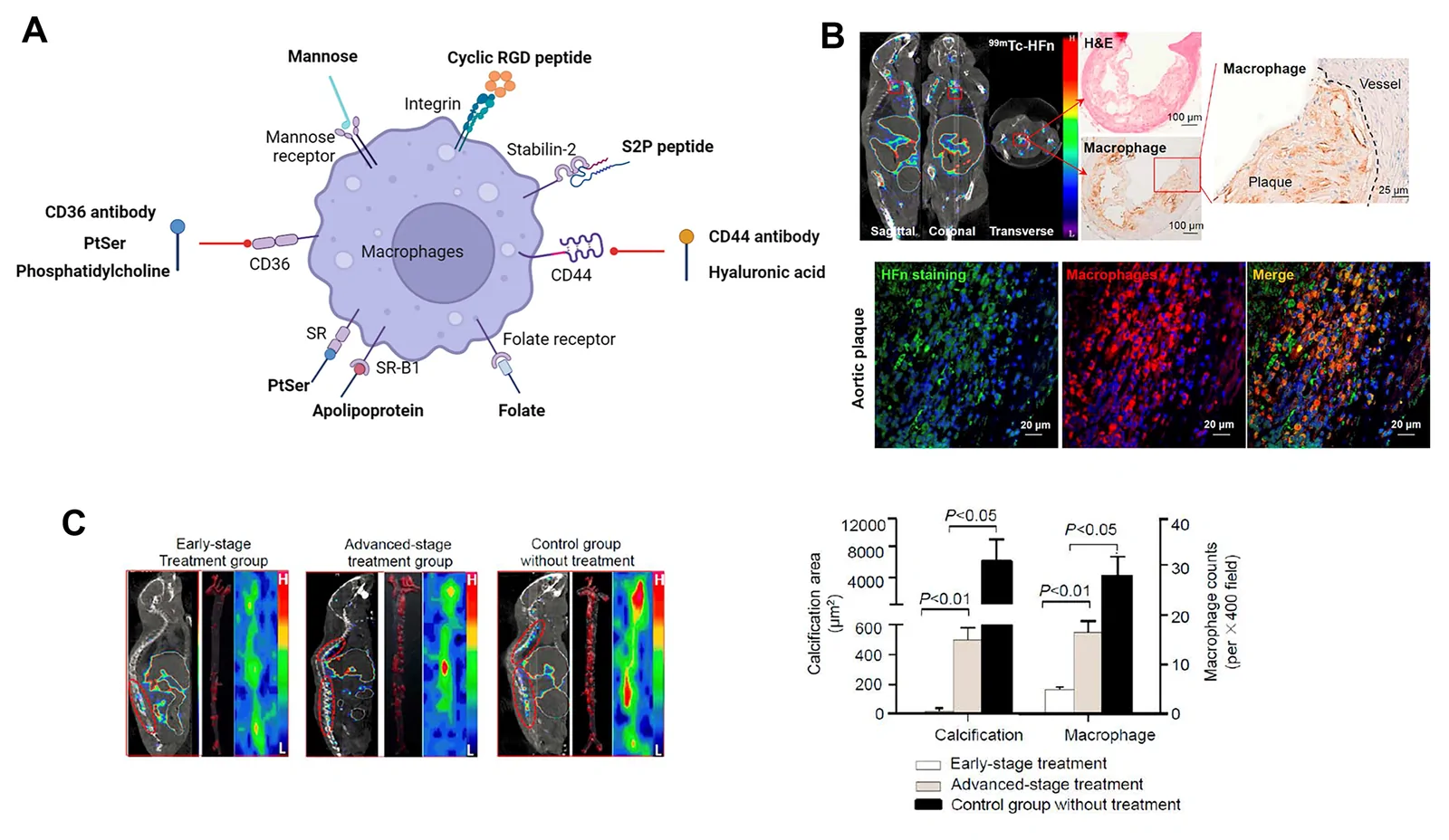
** Nanoparticles Targeted macrophages in atherosclerotic plaques via passive or active pathway.** (A) The surface molecules of macrophage for atherosclerotic active targeting. Reproduced with permission from Ref. 77. Copyright 2022, Oxford University Press. (B) Histology and immunofluorescent staining analysis of ^99m^Tc-HFn that demonstrated HFn was colocalized with macrophages within plaques. (C) Monitoring the dynamic inflammation changes of atherosclerotic plaques through ^99m^Tc-HFn SPECT-CT imaging. Reproduced with permission from Ref. 84. Copyright 2018, American Chemical Society.

**Figure 4:**
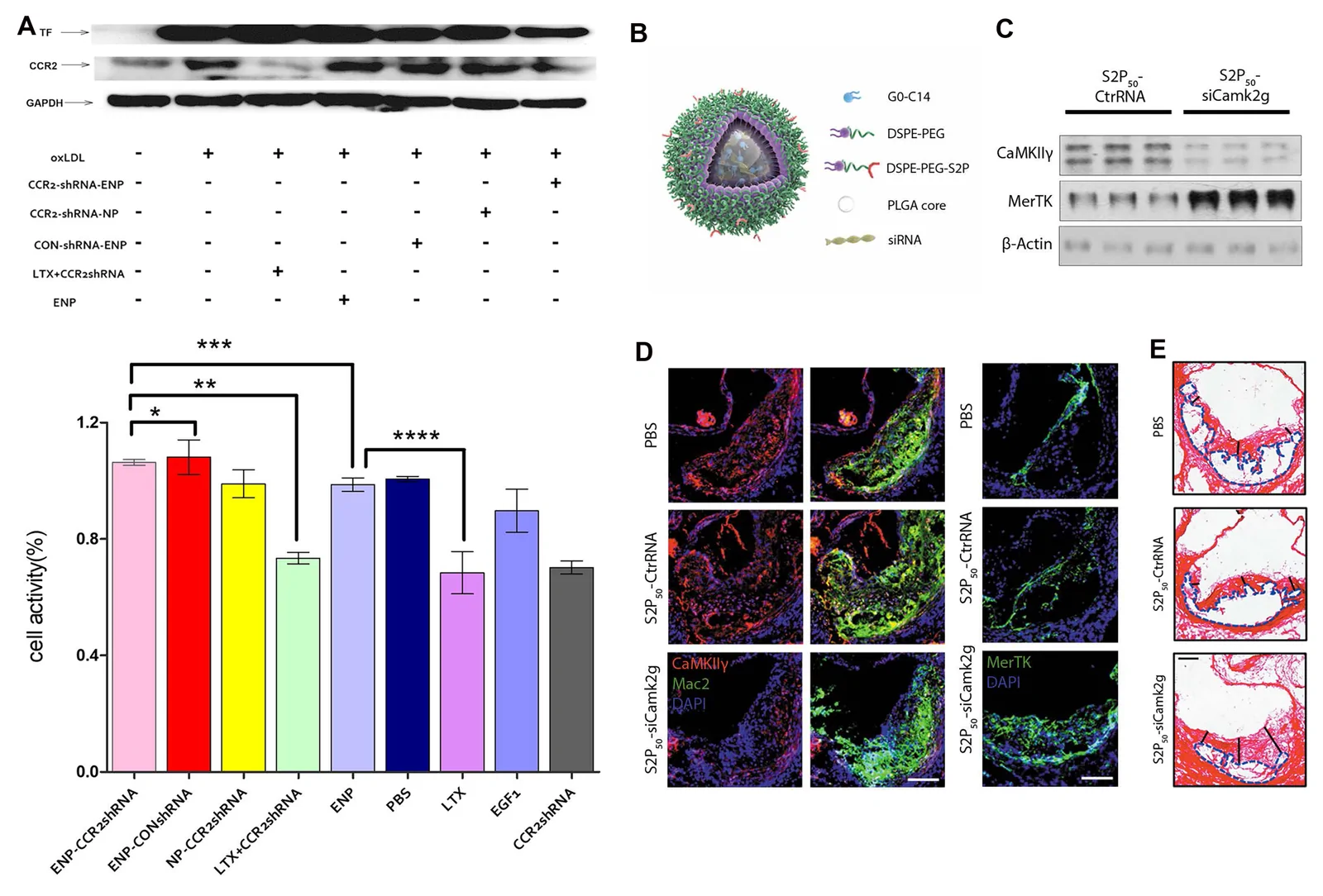
** PLGA-based nanomaterials targeting macrophage for AS therapy.** (A) CCR_2_ /TF protein expressions in Raw 264.7 cells and the cellular activity of Raw 264.7 cells after treatment with CCR_2_-shRNA-loaded ENPs or other control groups. Reproduced with permission from Ref. 88. Copyright 2020, Springer Nature. (B) Schematic diagram of PLGA-coated siRNA/C_0_-C_14_ complex. (C) Silencing efficiency of PLGA-coated siRNA/C_0_-C_14_ complex in cultured macrophages. (D) Silencing efficiency of PLGA-coated siRNA/C_0_-C_14_ complex in lesional macrophages of WD-fed Ldlr^-/-^ mice. (E) Aortic roots from the mice stained with picrosirius red. Reproduced with permission from Ref. 89. Copyright 2020, AMER ASSOC ADVANCEMENT SCIENCE.

**Figure 5:**
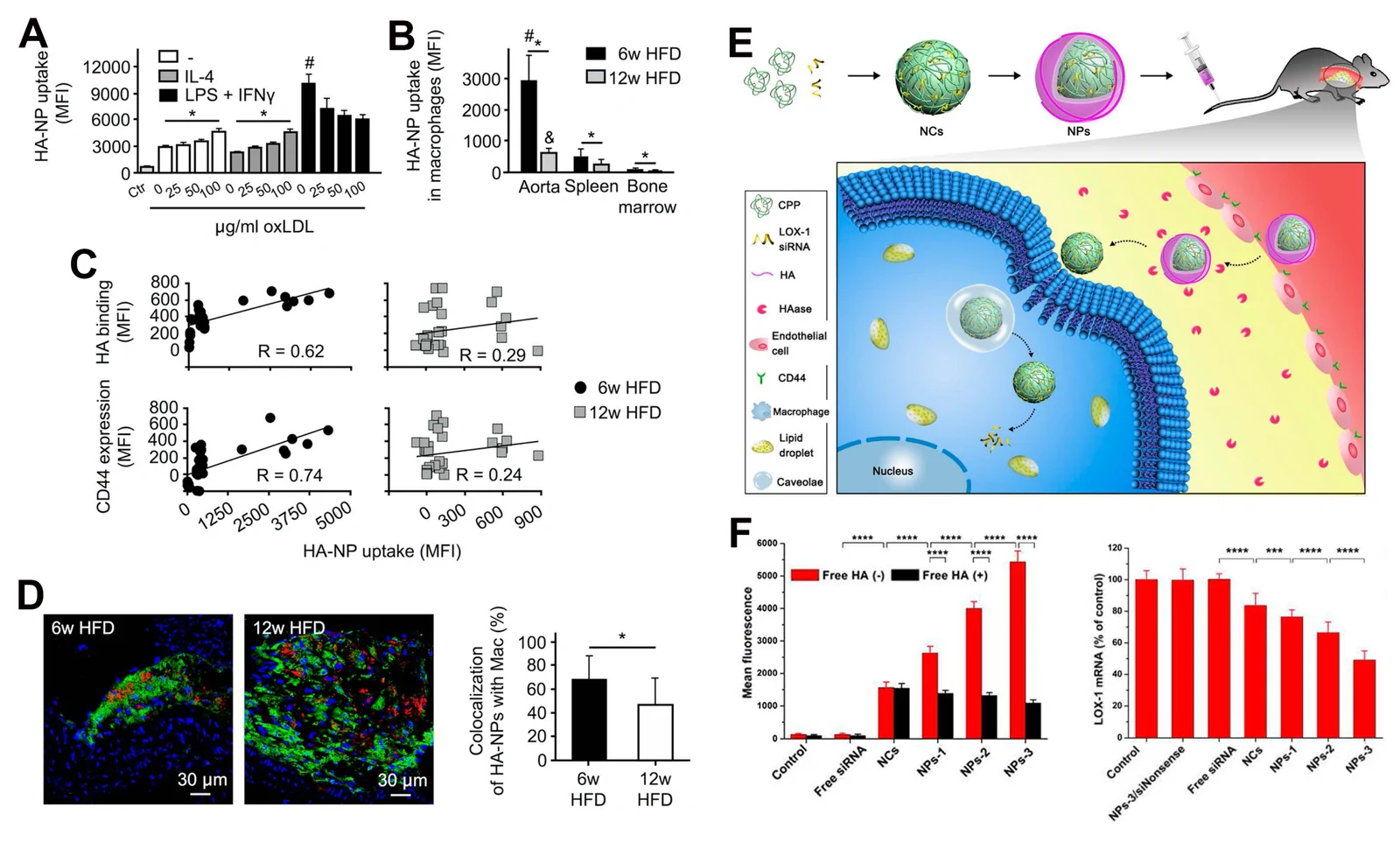
** HA-based nanomaterials targeting macrophage for AS therapy.** (A) Cellular uptake of Cy5.5-labeled HA NPs in three phenotypes of bone-marrow-derived macrophages measured by flow cytometry. (B) Uptake efficacy of Cy7-labeled HA NPs in aortic, splenic, and bone marrow macrophages measured by flow cytometry. (6w HFD: mice fed with a high-fat diet for 6 weeks; 12w HFD: mice fed with a high-fat diet for 12 weeks). (C) The relation between the uptake efficacy of HA NPs and expression of CD44 receptor. (D) Selectivity of HA-NPs toward plaque-associated macrophages (Cy5.5-labeled HA NPs are shown in red, CD68 staining of macrophages is green, and cell nuclei are blue). Reproduced with permission from Ref. 94. Copyright 2017, American Chemical Society. (E) Schematic illustration of HA-Coated (CPPs)/siRNA NPs for targeted gene delivery to plaques macrophages. (F) Cellular uptake and gene-silencing efficiency of various HA-Coated (CPPs)/siRNA NPs in THP-1-derived macrophages. Reproduced with permission from Ref. 99. Copyright 2018, American Chemical Society.

**Figure 6:**
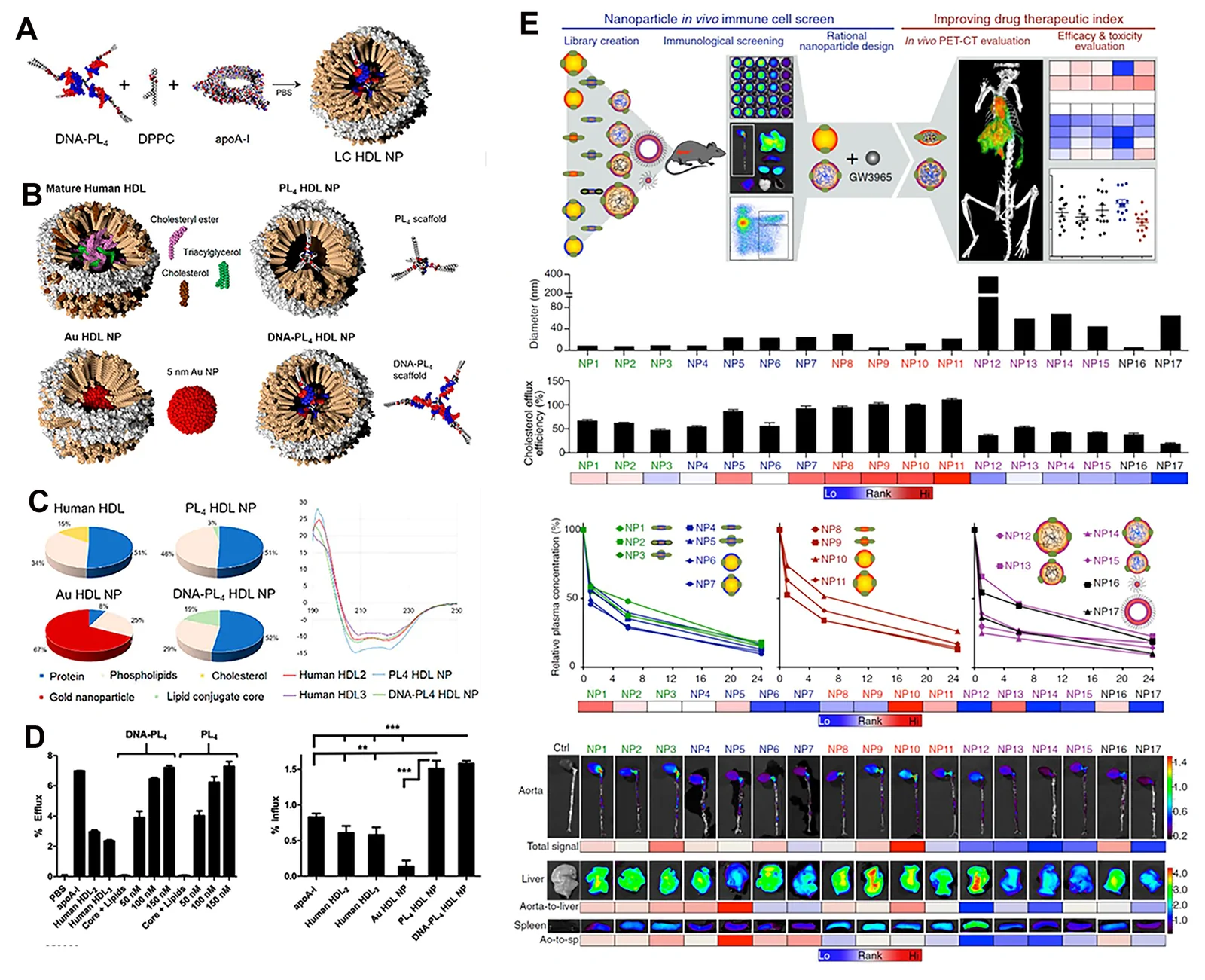
** rHDL-based nanomaterials targeting macrophage for AS therapy.** (A) Schematic illustration for LC HDL NP assembly. (B) Schematic illustration of natural and synthetic HDLs. (C) Composition and circular dichroism spectra of natural and synthetic HDLs and human HDLs. (D) Efflux and influx efficiency of synthetic HDLs and human HDLs. Reproduced with permission from Ref. 111. Copyright 2019, American Chemical Society. (E) Study design and evaluation of the 17 rHDL NPs library, including the size, cholesterol efflux capacity in primary macrophages, plasma concentration in Apoe^-/-^ mice fed with 12-wk high-cholesterol diet, and biodistribution. Reproduced with permission from Ref. 114. Copyright 2016, National Academy of Sciences (NAS).

**Figure 7:**
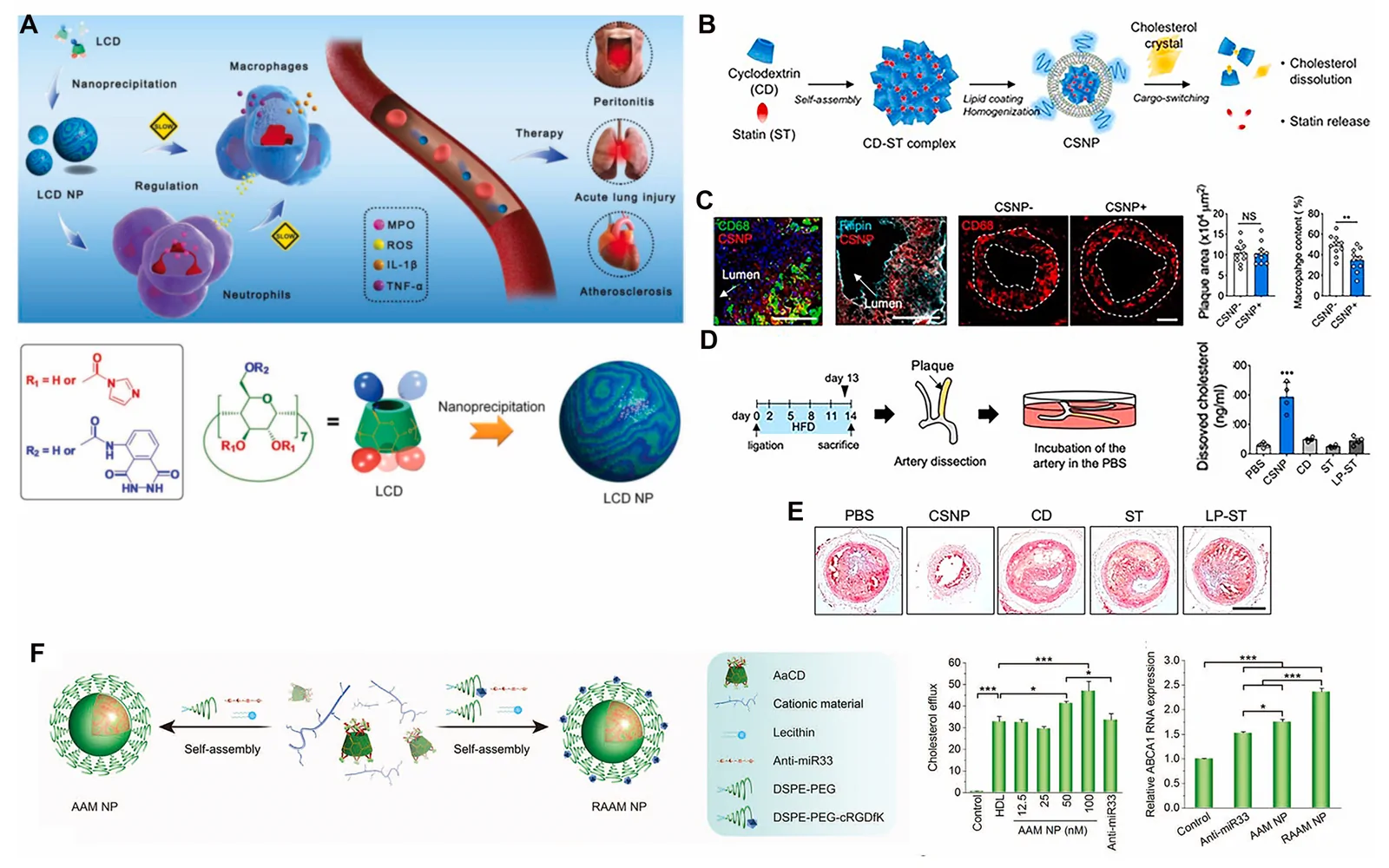
** Macrophage targeting CD-based nanomaterials for AS therapy.** (A) Schematic illustration of the nanoparticles using *β*-cyclodextrin and their anti-inflammatory role. Reproduced with permission from Ref. 121. Copyright 2019, WILEY-VCH. (B) Schematic illustration of CSNP preparation and cargo-switching. (C) Fluorescence images of the carotid artery sections after macrophage and/or cholesterol staining (macrophage, cholesterol, and nucleus were labeled by anti-CD68 antibody, Filipin, and Hoechst, respectively) and quantitative analysis. (D) Schematic illustration of *ex vivo* cholesterol dissolution assay and quantitative analysis of cholesterol content. (E) Representative histological images of the carotid artery sections after Oil-Red-O staining. Reproduced with permission from Ref. 123. Copyright 2020, American Chemical Society. (F) Schematic illustration of the pH-responsive anti-miR33 AAM NP for targeted treatment of atherosclerosis, as well as the cholesterol efflux and ABCA1 mRNA levels from RAW264.7 cells treated with anti-miR33-based different formulations. Reproduced with permission from Ref. 126. Copyright 2020, WILEY-VCH.

**Figure 8:**
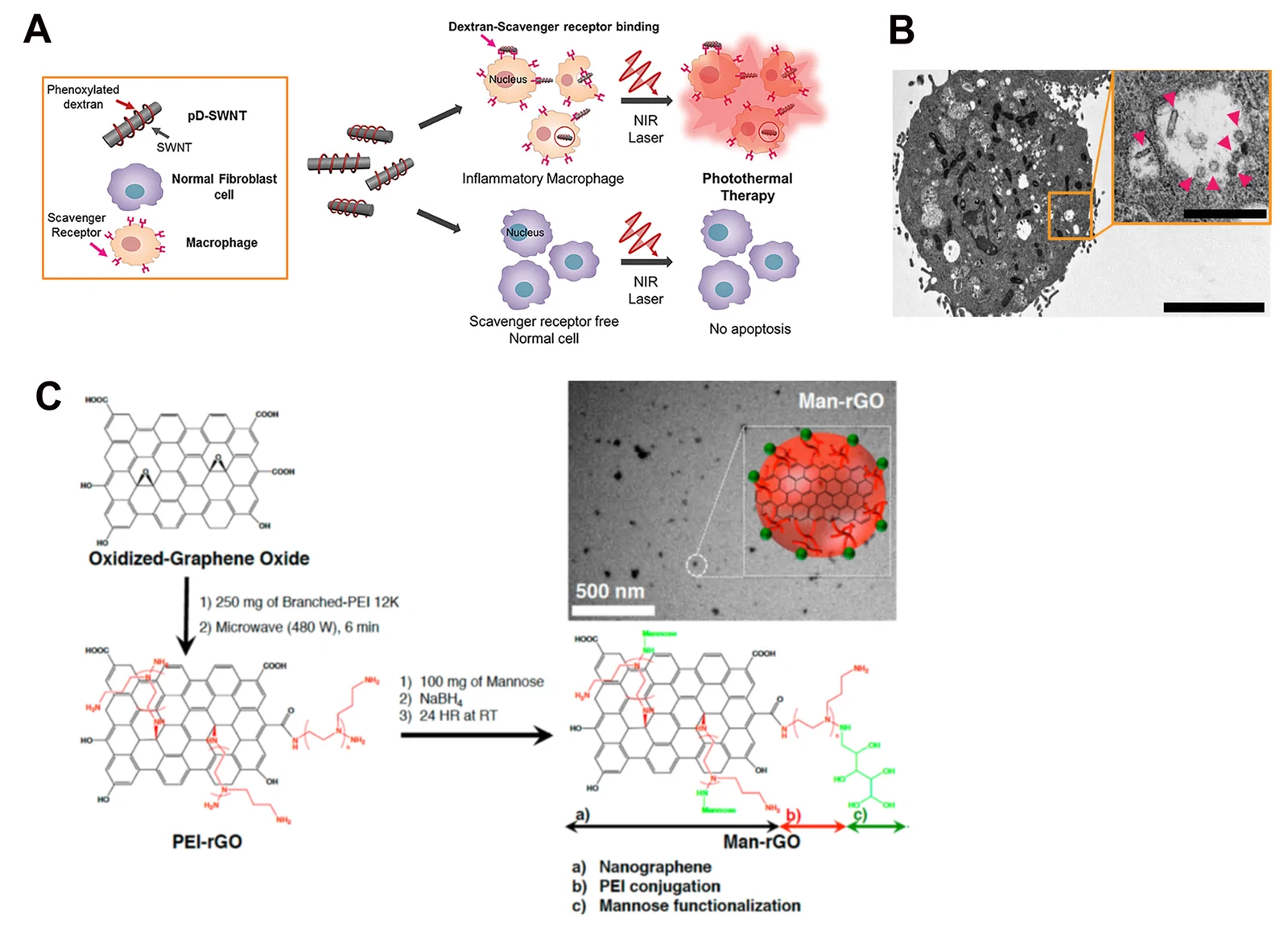
** Macrophage targeting SWNTs-based nanomaterials for AS therapy.** (A) Schematic illustration of the phenoxylated dextran modified SWNTs and the photothermal therapy of phenoxylated dextran modified SWNTs that specifically target against macrophages. (B) transmission electron microscopy (TEM) images of RAW 264.7 cells following incubation with pD-SWNTs. The main image features a scale bar of 5 μm, and the inset corresponds to a scale bar of 500 nm. Reproduced with permission from Ref. 128. Copyright 2016, WILEY-VCH. (C) A schematic representation of the preparation process for Man-rGO, and a TEM image of the resulting Man-rGOs. Reproduced with permission from Ref. 129. Copyright 2015, American Chemical Society.

**Figure 9:**
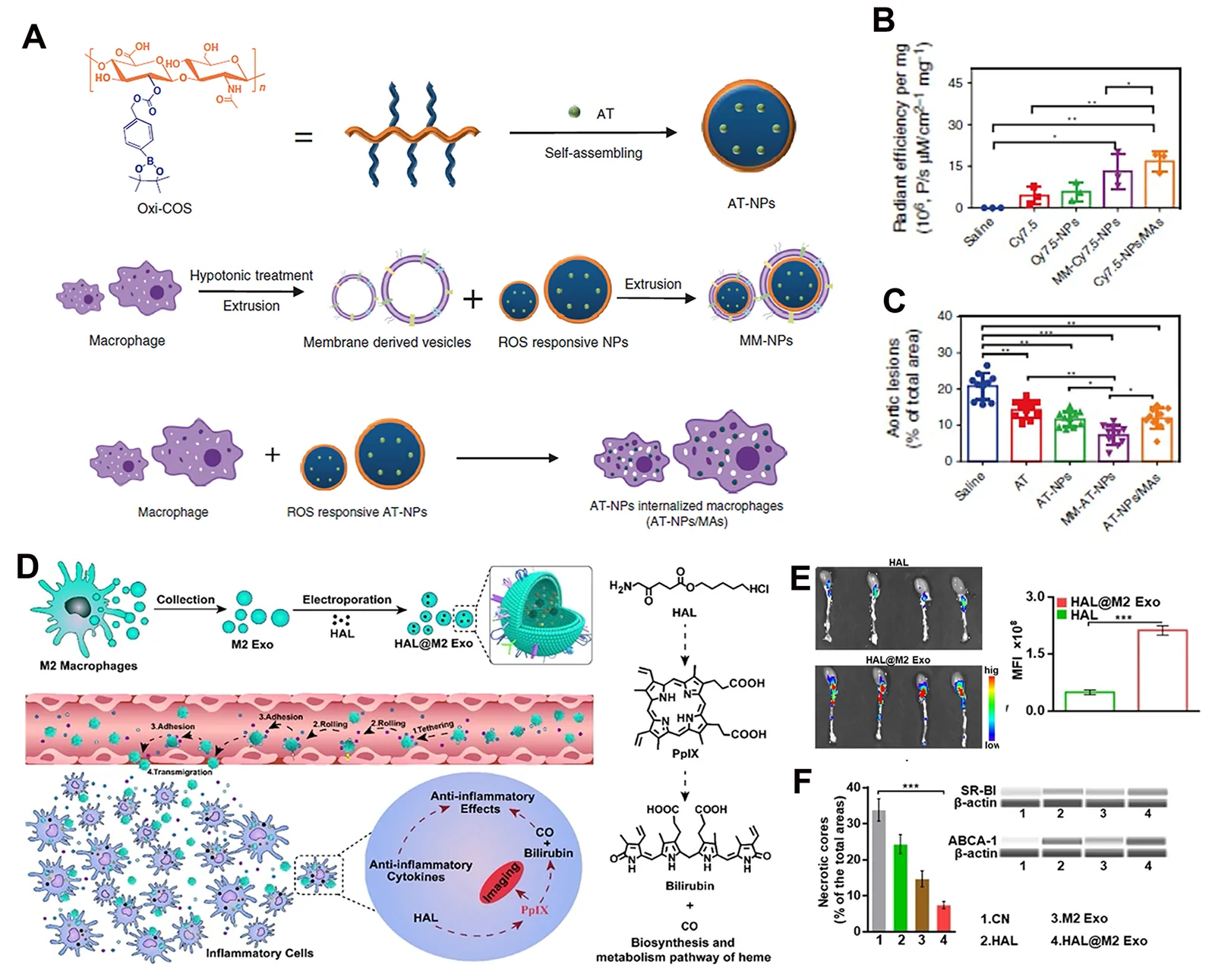
** Macrophage targeted Biomimetic nanomaterials for AS therapy.** (A) Schematic illustration of the preparation of AT-NPs, MM-AT-NPs and AT-NPs/MAs. (B) Quantitative analysis of fluorescent signal of Cy7.5-labeled AT-NPs, MM-AT-NPs and AT-NPs/MAs in aorta tissues. (C) Quantitative analysis of lesion area after different treatment in aorta tissues. Reproduced with permission from Ref. 140. Copyright 2020, Springer Nature. (D) Schematic illustration of HAL@M2 Exo and its anti-AS mechanism. (E) Fluorescence imaging assay confirmed the plaque targeting abilities of HAL@M2 Exo. (F) The necrosis area statistics and expression analyses of ABCA-1 and SR-BI after different treatments. Reproduced with permission from Ref. 142. Copyright 2020, WILEY-VCH.

**Figure 10:**
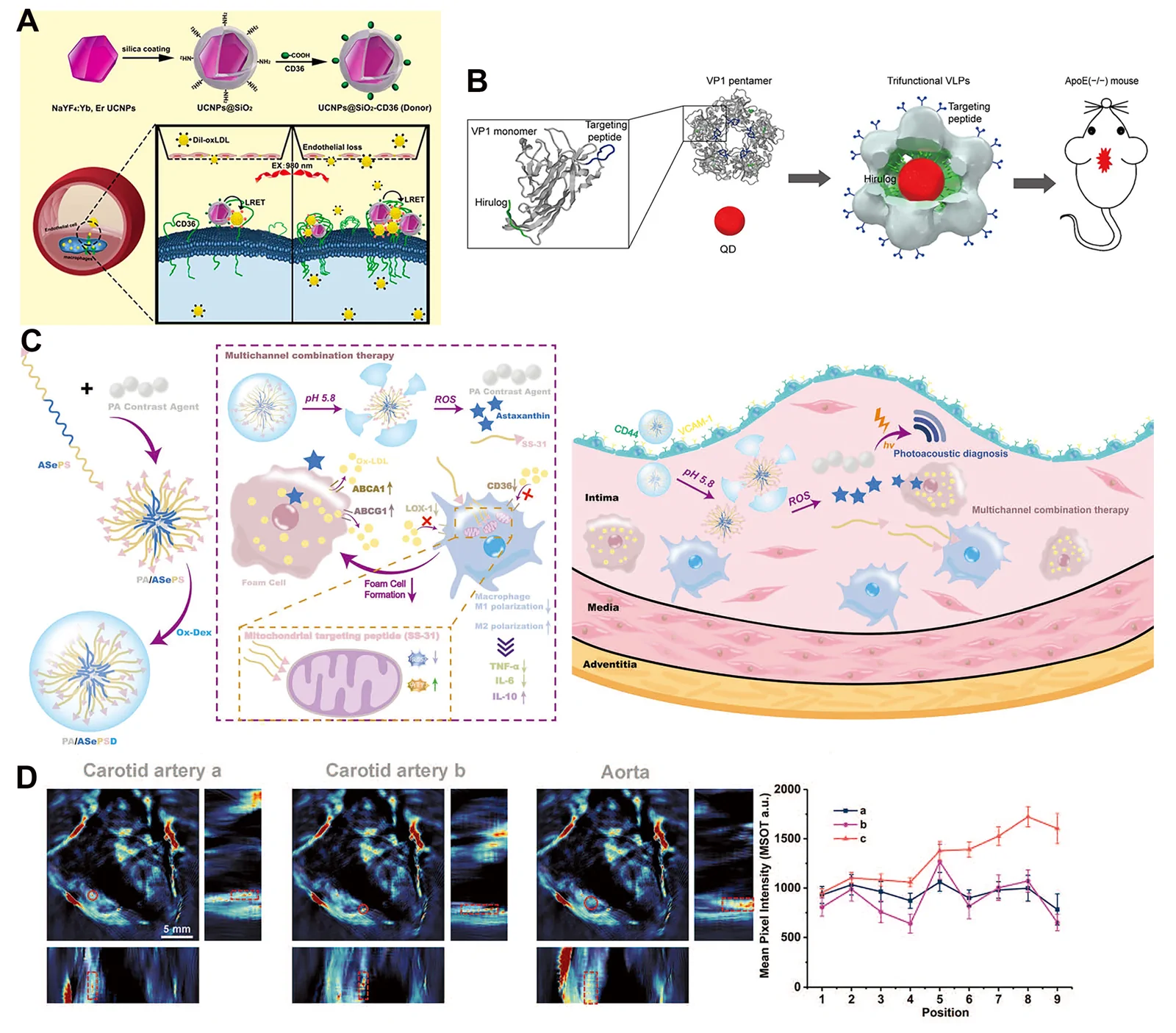
** Nanomaterials as contrast agents for AS diagnosis.** (A) Schematic illustration of UCNFP@SiO2-CD36 and the imaging of CD36 activation and CD36-oxLDL binding on a living via the interaction between UCNFP@SiO2-CD36 and DiI-oxLDL. Reproduced with permission from Ref. 165. Copyright 2019, American Chemical Society. (B) Schematic illustration of the structure SV40 VLPs bearing three targeting peptides. Reproduced with permission from Ref. 169. Copyright 2016, American Chemical Society. (C) Illustration of a dual-response PMeTPP-MBT nanoplatform loaded with a photoacoustic agent PA/ASePSD and equipped with cascade targeting for atherosclerosis photoacoustic imaging and multichannel combination therapy. (D) Photoacoustic signal distribution and quantitative photoacoustic intensity data of the aorta and carotid arteries in ApoE-/- mice treated with PA/ASePSD. Reproduced with permission from Ref. 172. Copyright 2023, WILEY-VCH.

**Figure 11:**
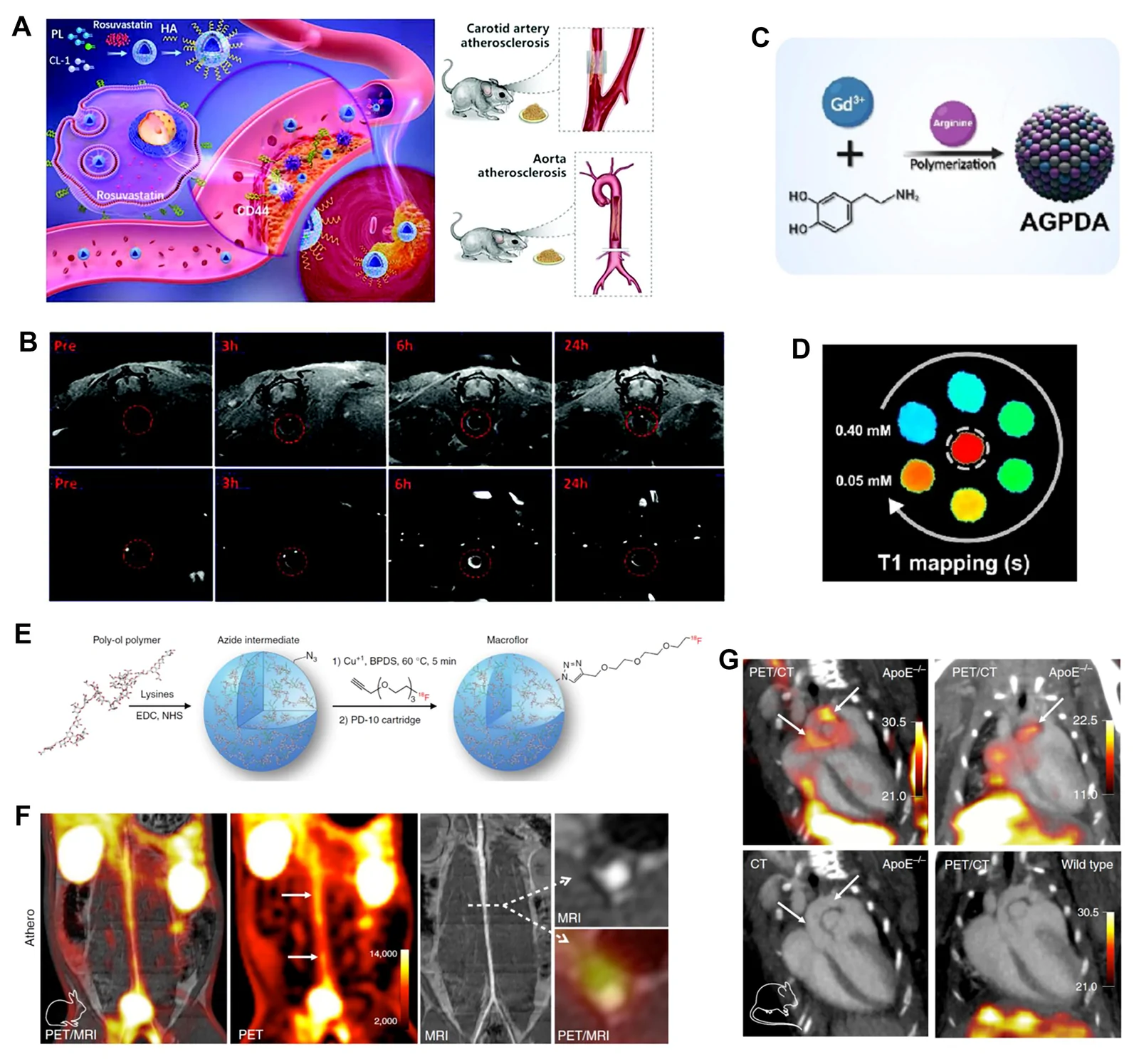
** Nanomaterials carrying contrast agents for AS diagnosis.** (A) Illustration of the diagnosis and treatment mechanism of HA-CC. (B) MRI of atherosclerotic aorta and quantitative analysis after addition of HA-CC-Gd. Reproduced with permission from Ref. 96. Copyright 2023, WILEY-VCH. (C) Schematic illustration of AGPDA nanoparticles. (D) Color map of T1 relaxation at various Gd concentrations of AGPDA nanoparticles. Reproduced with permission from Ref. 180. Copyright 2025, American Chemical Society. (E) Illustration of the synthesis and radiolabelling of ^18^F-Macroflor. (F) PET/MR imaging in atherosclerotic rabbits treated with ^18^F-Macroflor. (G) PET/MR imaging in ApoE^ -/-^ mice treated with ^18^F-Macroflor and wild-type control mice. Reproduced with permission from Ref. 182. Copyright 2017, Springer Nature.

**Table 1:** Overview of macrophage-targeted nanomaterials for AS therapy.

Nanomaterials	Targeting strategy	Therapy mechanism	Animal model	Ref
PLGA-*b*-PEG NPs	Passive target	Promotion of cholesterol efflux from macrophages	*Ldlr^-/-^* mouse fed an atherogenic western diet (WD)	46
Nanomedicine (CEZP) comprises PLGA NPs encapsulated within a metal-organic framework shell	Passive target	Restoration of efferocytic capacity of macrophages; Promotion of cholesterol efflux from macrophages; Anti-oxidation	ApoE^-/-^ mice fed a high-fat diet	85
NPs comprises cationic lipid-like molecule G_0_-C_14_ and PLGA-PEG	Mannose receptor	Anti-inflammatory therapies	Ldlr^-/-^ mouse fed an atherogenic western diet (WD)	82
EGFP-EGF1-conjugated PLGA NPs	Having a specific affinity to tissue factor (TF)	Reducing macrophage recruitment	---	88
NPs comprises cationic lipid-like molecule G0-C14, DSPE-PEG and PLGA-PEG	S_2_P peptide functionalization	Restoration of efferocytic capacity of macrophages	Ldlr^-/-^ mouse fed an atherogenic western diet (WD)	89
NPs comprises cationic lipid-like molecule G_0_-C_14_, thienothiadiazole-based near-infrared-II dye and PLGA-PEG	S_2_P peptide functionalization	Restoration of efferocytic capacity of macrophages	Ldlr^-/-^ mouse fed an atherogenic western diet (WD)	90
HA-guided cerasomes	HA functionalization	Anti-inflammatory therapies	Apoe^-/-^ mice fed with a high-fat diet	96
HA-modified hybrid macrophage membrane-liposome NPs	HA functionalization	Modulation of Lipid Metabolism and Phenotype of Macrophages	ApoE^-/-^ mice fed with a high-methionine diet	97
pH-sensitive HA-based NPs	HA functionalization	Inhibiting the inflammatory response	ApoE^-/-^ mice fed with a high-fat diet	98
HA-Coated CPPs/siRNA NPs	HA functionalization	Inhibition of macrophage proliferation	ApoE^-/-^ mice fed with a high-fat diet	99
rHDL NPs	apo A-i with high affinity for SR-B1	Inhibiting the inflammatory response	apoE-KO mice fed with a high-fat diet	109
sHDL NPs	apo A-i with high affinity for SR-B1	Promotion of cholesterol efflux from macrophages	ApoE-deficient mice fed with an atherogenic diet (HFHC, 21% fat, 34% sucrose, and 0.2% cholesterol, Harlan, T.D. 88,137)	110
Dual targeting Multifunctional rHDL-mimicking core-shell nanoplatform	CD36 ligand phosphatidylserine (PS)	Promotion of cholesterol efflux from macrophages, Inhibition of macrophage proliferation	ApoE^-/-^ mice fed with a high-fat diet	112
Nanoplatform fabricated by cationic dendritic lipopeptide (G_2_K), PLGA, DSPE-PEG-S_2_P and DSPE-PEG-β-CD	S_2_P peptide functionalization	promoting cholesterol efflux and reducing inflammation	ApoE^-/-^ mice fed with a high-fat diet	119
luminol-conjugated β-cyclodextrin NPs	Passive target	inhibiting neutrophil-mediated inflammatory macrophage recruitment	ApoE^-/-^ mice fed with an atherogenic western diet	121
β-CD decorated macrophage-liposome conjugate (MP-QT-NP)	Passive target	promoting cholesterol efflux and reducing inflammation	ApoE^-/-^ mice fed with a high-fat diet	122
Tempol and phenylboronic acid pinacol ester conjugated β-cyclodextrin NPs	Passive target	Anti-oxidation therapies	ApoE^-/-^ mice fed with a high-fat diet	125
Cargo-switching methyl-β-cyclodextrin NPs	Passive target	reducing the content of plaque cholesterol and macrophages	ApoE^-/-^ mice fed with a Paigen’s high-fat diet	123
CD-derived pH-responsive NPs	cRGDfK functionalization	reducing the content of plaque cholesterol, Modulation of the Phenotype of Macrophages	ApoE^-/-^ mice fed with the Western diet	126
Single-walled carbon nanotubes (SWNTs)	Passive target	Promoting macrophage efferocytosis	ApoE^-/-^ mice fed with the Western diet	130,131
Apoptotic body biomimic liposome (AP-Lipo)	Passive target	upregulating anti-inflammatory macrophages number	ApoE^-/-^ mice fed with a high-fat diet	136
M1 macrophage membrane-coated nanoprodrug	Passive target	inhibiting macrophage phagocytosis	ApoE^-/-^ mice fed with a high-fat diet	137
Macrophage membrane (MM) coated rapamycin-loaded PLGA NPs	Passive target	inhibiting macrophage phagocytosis	ApoE^-/-^ mice fed with a high-fat diet	138
M2 macrophage membrane-derived nanovesicles co-fused with lipids	Passive target	suppressing inflammation, reducing the proliferation of macrophages	ApoE^-/-^ mice fed with a high-fat diet	139
Macrophage membrane coated ROS-responsive NPs	Passive target	suppressing local inflammation and inflammatory cytokines sequestration	ApoE^-/-^ mice fed with a high-fat diet	140
Molecularly engineered M2 macrophage-derived exosomes	Passive target	suppressing inflammation	ApoE^-/-^ mice fed with a cholesterol-rich diet	142

**Table 2:** Overview of macrophage-targeted nanomaterials for AS diagnosis.

Nanomaterials	Targeting strategy	Imaging modality	Animal model	Ref
Fe-PFH PLGA/chitosan (CS)- dextran sulfate (DS) NPs	DS targeting class A scavenger receptors (SR-A)	MRI	ApoE^-/-^ mice fed with a high-fat diet	158
PLGA nanoparticles containing Fe_3_O_4_ and perfluoropentane, and coated with peptides PP1 and cyclic RGD	PP1 and cyclic RGD targeting peptides	US/MR	ApoE^-/-^ mice fed with a western diet	159
PP1-Au@GSH@Gd nanoclusters	PP1targeting peptides	MR/fluorescence molecular imaging	ApoE^-/-^ mice fed with a high-fat diet	164
CD36-antibody-modified, SiO_2_-coated upconversion luminescent NPs (UCNPs)	CD36-antibody	Fluorescence imaging	---	165
anti-MARCO NaGdF_4_:Yb,Er@NaGdF_4_ upconversion NPs (UCNPs)	Polyclonal MARCO antibody	Optical/MRI dual-modality imaging	ApoE^-/-^ mice fed with a high-fat diet	166
Trifunctional Simian virus 40 (SV_40_)-based NPs encapsulated near-infrared quantum dots	CGNKRTRGC peptide, Hirulog peptide, fusion protein Hir-M-VP1	Fluorescence imaging	ApoE^-/-^ mice fed with a high-fat diet	169
π-conjugated polymer (PMeTPP-MBT) based nanoplatform	SS-31 peptide	PA imaging	ApoE^-/-^ mice fed with a high-fat diet	172
Semiconducting conjugated polymers contained theranostic platform encapsulating si-Olfr_2_	Passive target	PA imaging	ApoE^-/-^ mice fed with high-cholesterol diet	173
Macrophage-targeted, enzyme-triggered fluorescence switch-on liposome	Phosphatidylserine functionalization	Fluorescence imaging	ApoE^-/-^ mice fed with a high-fat diet	176
HA-guided cerasome nano-formulation loaded with Gd	HA functionalization	MRI	ApoE^-/-^ mice fed with a high-fat diet	96
Polydopamine nanoparticles doped with arginine and gadolinium ions (AGPDAR-146a)	Phosphorothioate backbone-modification for targeting SR-A	MRI	ApoE^-/-^ mice fed with a high-fat diet	180
^18^F-Macroflor modified polyglucose NPs	Passive target	PET/ MRI	ApoE^-/-^ mice fed with a high-cholesterol diet	182
Ultrasmall USPIO functionalized with ^99m^Tc coordination and Annexin V	Annexin V functionalization for targeting apoptotic macrophages	SPECT/MRI	ApoE^-/-^ mice fed with a high-fat diet	184

## Abbreviations

CVDs: cardiovascular diseases
AS: Atherosclerosis
ROS: reactive oxygen species
SMCs: smooth muscle cells
M-CSF: Monocyte colony-stimulating factor
GM-CSF: granulocyte-monocyte colony-stimulating factor
LXR: liver X receptor
ABCA1: ATP-binding cassette transporter A1
ApoE: apolipoprotein E
OX-LDL: Oxidized low-density lipoprotein
MMPs: matrix metalloproteinases
SRs: macrophage scavenger receptors
LOX-1: lectin-like oxidized LDL receptor-1
MARCO: macrophage receptor with collagenous structure
SDIO: dextran sulfate
DXS: dextran sulfate
AT: atorvastatin calcium
rHDL: reconstituted high density lipoprotein
sHDL: synthetic HDL
LCAT: lecithin cholesterol acyltransferase
mDNP: mannose-decorated dendrimeric nanoparticle
NLRP3: NACHT, LRR, and PYD domain protein 3
ATP: adenosine triphosphate
HA: hyaluronic acid
O_2_^.-^: superoxide radicals
H_2_O_2_: hydrogen peroxide
HO.: hydroxyl radicals
ONOO^-^: peroxynitrit
ZOL: zoledronic acid
PB: probucol
CD: cyclodextrin
ECM: extracellular matrices
RAP: rapamycin
MPS: mononuclear phagocyte system
FR: folate receptor
TfR1: transferrin receptor 1
HFn: Human heavy-chain ferritin
SPECT: single-photon-emission computed tomography
CT: computed tomography
PPARγ: peroxisome proliferator–activated receptor-γ
TF: tissue factor
CaMKII: calcium-activated kinase
HAase: hyaluronidase
RES: reticuloendothelial system
CPPs: cell-penetrating peptides
ADA: adamantane
QT: quercetin
PEI: polyethyleneimine
GO: graphene oxide
SWNTs: single-walled carbon nanotubes
NIR: near-infrared radiation
SiRP*α*: signal regulatory protein-*α*
TPI: tyrosine phosphatase inhibitor
HAL: hexyl 5-aminolevulinate hydrochloride
MRI: magnetic resonance imaging
PET: positron emission tomography
PAI: photoacoustic imaging
FI: fluorescence imaging
OPN: osteopontin
SPR: surface plasmon resonance
UCNPs: Upconversion nanoparticles
LRET: luminescence resonance energy transfer
QDs: Quantum dots
SV40: Simian virus 40
PS: phosphatidylserine
RST: rosuvastatin
